# A self-replicating artificial module-genome that generates bacterial chromosome replication system *in vitro*

**DOI:** 10.1093/nar/gkag663

**Published:** 2026-07-06

**Authors:** Yuta Yamagishi, Yoshiki Sonoyama, Naoki Kawakami, Tomonori Hasebe, Masayuki Su’etsugu

**Affiliations:** Department of Life Science, College of Science, Rikkyo University, 3-34-1 Nishi-Ikebukuro, Toshima-ku, Tokyo 171-8501, Japan; Department of Life Science, College of Science, Rikkyo University, 3-34-1 Nishi-Ikebukuro, Toshima-ku, Tokyo 171-8501, Japan; Department of Life Science, College of Science, Rikkyo University, 3-34-1 Nishi-Ikebukuro, Toshima-ku, Tokyo 171-8501, Japan; Department of Life Science, College of Science, Rikkyo University, 3-34-1 Nishi-Ikebukuro, Toshima-ku, Tokyo 171-8501, Japan; Department of Life Science, College of Science, Rikkyo University, 3-34-1 Nishi-Ikebukuro, Toshima-ku, Tokyo 171-8501, Japan

## Abstract

Autonomous self-reproduction is a major goal of bottom-up synthetic biology aimed at building artificial cells. This requires that the genome be replicated by its self-encoded replication machinery. While the reconstituted *Escherichia coli* chromosomal replication system, termed the Replication-Cycle Reaction (RCR) system, offers a promising platform for genome-scale replication, its generation from genetic information has not yet been achieved. Here we show that a 53 kb circular DNA, termed RCR module-genome, encoding all 26 RCR proteins, can self-replicate in a one-pot reaction when expressed using the protein synthesis using recombinant elements (PURE) system. We first built a prototype of the RCR module-genome and then optimized reaction conditions and solved expression bottlenecks to achieve robust self-replication. This artificial module-genome supports more than 28 doublings of recursive self-replication. This system, termed PRIMES (PURE-driven RCR for *In-vitro* Module-gEnome Self-replication), represents a milestone toward constructing self-reproducing artificial cells.

## Graphical Abstract

**Graphical Abstract:**
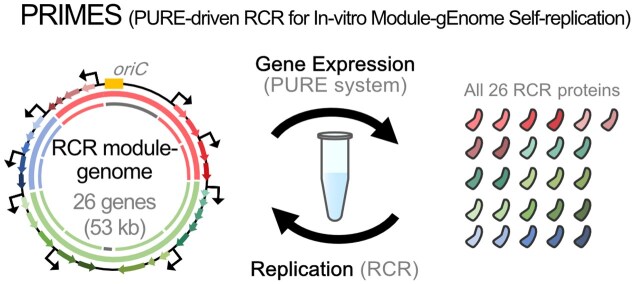

## Introduction

Research into artificial cell systems aims to elucidate the fundamental design principles of life by reconstituting biological systems from defined molecular components [1–5]. A long–standing goal is to construct artificial cells capable of autonomous self–reproduction. In living systems, self–reproduction is achieved by a system in which all cellular constituents are regenerated under the control of genomic information. This process rests on the central dogma—transcription, translation, and replication. Specifically, genes encoding the central–dogma machinery are expressed through transcription and translation, after which replication factors act to duplicate the genome itself. Accordingly, the next critical step is to reconstitute a self–reproducing central–dogma module *in vitro*.

Recently, self–reproduction of the transcription and translation machinery has advanced through *in vitro* reconstitution. The PURE system, an *in vitro* transcription–translation reconstitution system, is reconstituted using individually purified components derived from *Escherichia coli* [6]. Self–reproduction of PURE system components has been demonstrated for transfer RNAs (tRNAs) [7–10], aminoacyl–tRNA synthetases [11, 12], and translation factors [13, 14], which are expressed from DNA templates encoding the respective genes. Nevertheless, achieving ribosome self–reproduction with PURE–expressed products remains challenging. To date, partial ribosome biogenesis has been reported [15–17]. In cell–free systems based on *E. coli* extracts, ribosomal biogenesis has also been observed at modest levels [18].

In the context of DNA replication, self-replicating systems have been developing rapidly in which the replication machinery synthesized via the PURE system replicates the template DNA encoding it. Previously reported self-replicating DNA systems have utilized phi29-derived DNA polymerase as their replication machinery. Ichihashi and colleagues achieved self-replication of circular DNA by combining rolling-circle replication with Cre recombinase-mediated circularization [19, 20]. Similarly, Danelon and colleagues demonstrated self-replication of a ∼3 kb linear DNA template encoding four proteins required for phi29 DNA replication [21, 22]. These phage-based systems represent pioneering achievements. The phi29 phage genome itself is approximately 19.3 kb [23], and self-replication of DNA templates of several kilobases has been demonstrated using this replication system [21, 22]. The construction of artificial cells, however, requires self-replication of larger DNA templates encoding numerous factors responsible for the central dogma, and thus demands a genome-scale replication platform.

The *E. coli* chromosomal replication system represents a promising platform for achieving self-replication of large genomes. *Escherichia coli* replicates its 4.6 Mb circular genome bidirectionally from *oriC*, with simultaneous leading and lagging strand synthesis [24, 25]. Replication terminates at the opposite terminus, followed by decatenation and supercoiling to regenerate templates for the next cycle [26]. This mechanism enables stable amplification of a large circular genome. Reconstitution of the *E. coli* chromosomal replication system has been achieved using purified proteins. The Replication-Cycle Reaction (RCR) system, comprising 26 proteins, has been established to execute the entire replication cycle, including initiation, elongation, termination, decatenation, and supercoiling [27]. Through autonomous, recursive repetition of this replication cycle, the RCR system achieves exponential amplification of circular DNA having *oriC* and has been shown to amplify up to 2 Mb [28]. To date, Fujiwara *et al*. demonstrated functional expression of 13 proteins for *E. coli* chromosomal replication in the PURE system [29]; however, full generation of all 26 RCR proteins has not yet been achieved.

**Figure 1.**
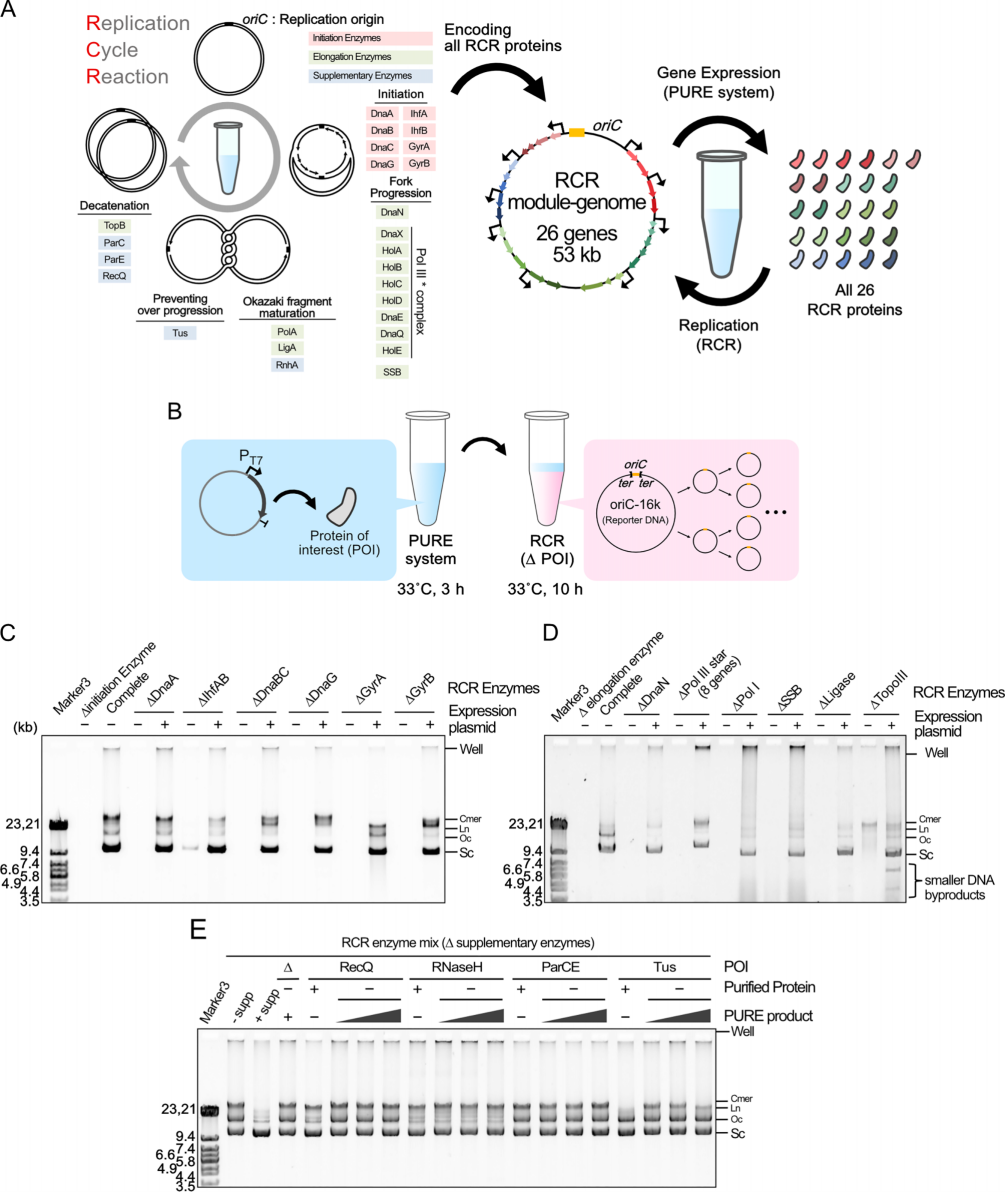
Functional validation of RCR proteins expressed in the PURE system. (**A**) Schematic of the RCR module-genome self-replication system. The RCR module-genome encodes all 26 RCR proteins and is replicated by its own expression products. (**B**) Schematic of the rescue assay. Each protein or protein complex of interest (POI) is expressed from a pET plasmid in the PURE system (3 h, 33°C) and added to an RCR reaction lacking only the POI (10 h, 37°C). Activity is assessed by amplification of a reporter plasmid (oriC-16k, 0.5 ng/µl; Supplementary Table 1). (**C–E**) Rescue assay results for each RCR protein group. Reaction products were separated by 1% agarose gel electrophoresis (60 V, 60 min) and stained with SYBR Green I. The leftmost lane shows a DNA size marker. Each lane shows a reaction with (+) or without (–) the indicated PURE-expressed protein. Bands indicate supercoiled (sc), open circular (oc), linear (ln), concatemer (Cmer), and replication intermediates in the well (Well). (**C**) Initiation enzymes. All initiation enzymes rescued RCR activity. Faint amplification without IhfAB suggests a stimulatory but nonessential role. (**D**) Elongation enzymes. All elongation enzymes rescued RCR activity. Concatemer amplification was observed in the TopB-lacking control. (**E**) Supplementary enzymes. DNA amplification occurred without supplementary enzymes, but their addition reduced byproducts such as concatemers and open circular DNA, increasing the supercoiled product. To examine these effects, their PURE system products were added at final concentrations of 1%, 3%, and 10%. Supp indicates supplementary enzymes.

In this study, we designed and constructed a 53 kb circular DNA, designated the “RCR module-genome”, which is a functional module containing a complete set of genes for the chromosomal replication cycle. Specifically, it encodes all 26 RCR proteins and *oriC* on a single circular DNA (Fig. 1A). To achieve self-replication, we established the PURE-RCR system, in which RCR proteins expressed via the PURE system drive the replication cycle. Through optimization of reaction conditions and gene expression levels, we achieved self-replication of the RCR module-genome. We termed this self-replication system PRIMES (PURE-driven RCR for *In-vitro* Module-gEnome Self-replication). We also demonstrate that the PRIMES system sustains five recursive reaction rounds of self-replication. This achievement represents a fundamental milestone toward the construction of artificial cells. The self-replicating RCR module-genome provides a platform for future efforts to integrate additional genetic modules required for autonomous cellular functions.

## Materials and methods

### DNA constructs

DNA sequences and detailed information for plasmids used in rescue assays are provided in Supplementary Table 1. DNA sequences and detailed information for constructs used in RCR module-genome assembly are provided in Supplementary Table 2 and polymerase chain reaction (PCR) fragment information is summarized in Supplementary Table 3. The pET plasmids encoding replication genes used for rescue assay were constructed as previously described [27]. To construct the RCR module-genome, we designed operon plasmids encoding replication gene groups organized by function. Each operon was designed with 2–5 genes under the control of a T7 promoter, followed by a highly efficient T7hyb10 terminator [30]. Most genes carry an optimized ribosome binding site (RBS) derived from the 5′ untranslated region of the pET21a vector. Complete sequences of operon plasmids are provided in GenBank format. All *oriC*-containing constructs carry two inward-facing *terB* sites flanking *oriC*, as described previously [31].

**Figure 2.**
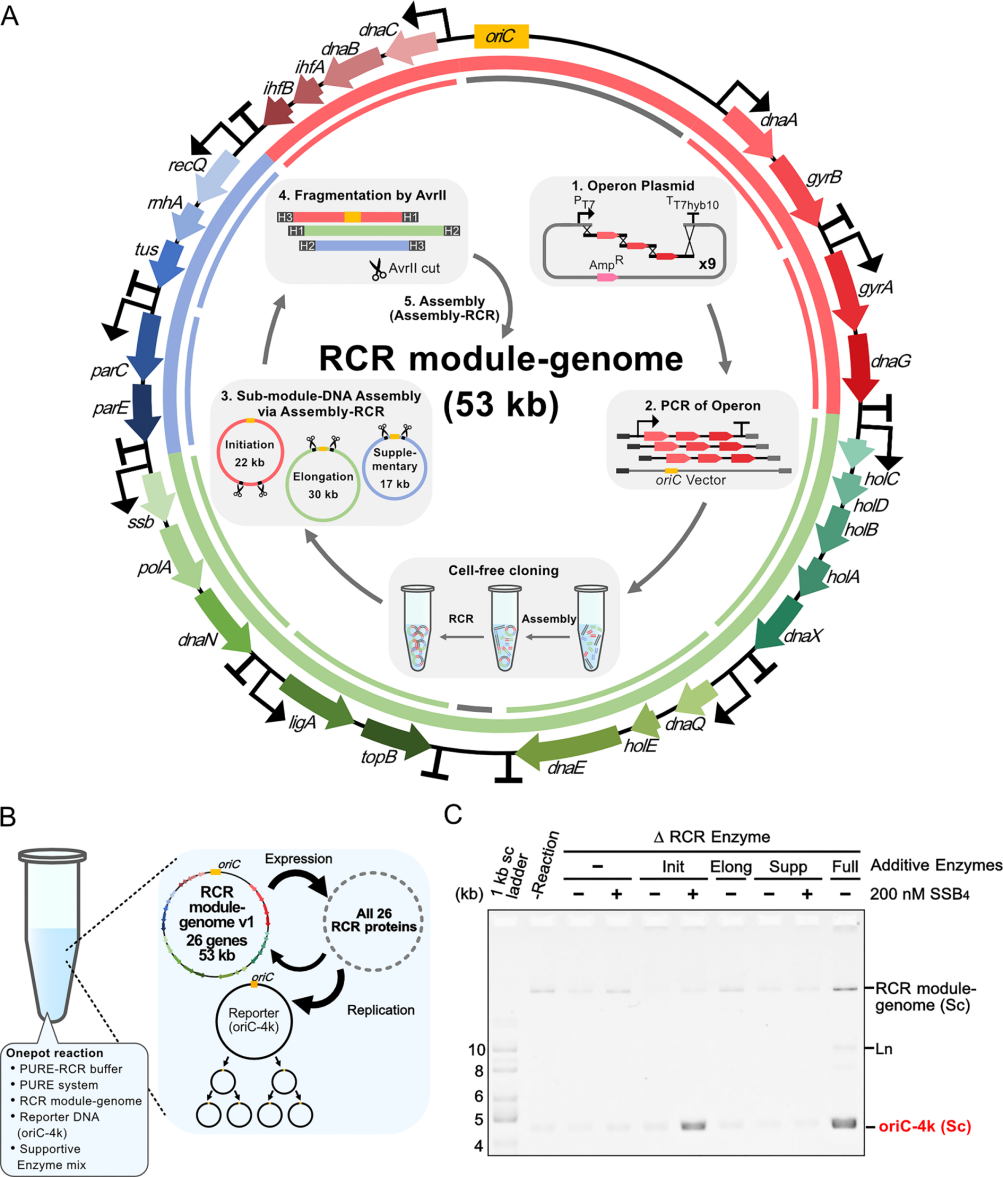
Construction and functional assessment of the RCR module-genome. (**A**) Scheme for the construction of the RCR module-genome. Nine operon plasmids were amplified by PCR and assembled into three sub-module-DNAs via the Assembly-RCR method. Each sub-module-DNA was then linearized with the restriction enzyme AvrII and assembled in a second Assembly-RCR using pre-designed homologous regions to construct the full-length 53 kb RCR module-genome. All DNA sequences used in this study are listed in Supplementary Table 1. (**B**) Schematic of the reporter assay to evaluate the activity of the RCR module-genome. The activity of the expression products from the RCR module-genome (100 pM) was assessed by the amplification of a 250 pM promoter-less reporter DNA (oriC-4k). (**C**) Agarose gel electrophoresis of the reporter assay results. After a 9-h incubation at 37°C, products were separated on a 1% agarose gel (60 V, 60 min) and stained with SYBR Green I. The reporter DNA was not amplified by the RCR module-genome expression products alone but required supplementation with purified initiation enzymes (1 × concentration) and SSB (200 nM). Init, Elong, and Supp indicate initiation enzymes, elongation enzymes, and supplementary enzymes, respectively.

The workflow for constructing sub-module-DNAs and the RCR module-genome is shown in Fig. 2A. Sub-module-DNAs were constructed using seamless assembly and the RCR method. Operon fragments were amplified from operon plasmids by PCR using KOD One^®^ PCR Master Mix (TOYOBO). PCR conditions were as follows: initial denaturation at 98°C for 2 min, followed by 25 cycles of 98°C for 10 s and 68°C for extension at 5 s/kb. PCR products were treated with DpnI at 37°C for 1 h to remove template DNA and purified using NucleoSpin^®^ Gel and PCR Clean-up (MACHEREY-NAGEL). The purified fragments, which have 40 bp overlaps at each end, were assembled using an *in vitro* recombination system equivalent to OriCiro Assembly Kit (OriCiro Genomics, Inc.). The assembly reaction was then subjected to RCR as previously described [27], containing 1 × RCR buffer I and II, 1 × replication enzyme (RE) mix, Exonuclease I (200 mU/μl, NEB), Exonuclease V (RecBCD, 200 mU/μl, NEB), RecG 60 nM, RecJ 0.5 U/μl, and ExoIII 60 mU/μl [32]. RCR selectively amplifies only circularized products containing *oriC*, yielding three sub-module-DNAs: initiation enzymes (22 kb, three operons), elongation enzymes (30 kb, four operons), and supplementary enzymes (17 kb, two operons).

The RCR module-genome was constructed by assembling these sub-module-DNAs using the seamless assembly reaction. The oriC-vector region (8 kb) was removed from the elongation enzymes and supplementary enzymes sub-module-DNAs by digestion with AvrII (NEB, R0174L). The initiation enzymes sub-module-DNA was also digested with AvrII to generate the fragment containing *oriC*. These linearized fragments were mixed and assembled based on pre-designed homologous sequences, then amplified via RCR to obtain the complete 53 kb RCR module-genome.

The amplified DNA was subjected to five-fold dilution with 1 × RCR buffer I and II and further incubated at 37°C for 15 min for finalization of replication intermediates. *Escherichia coli* HST08 Premium Electro-Cells (Takara) were transformed with the RCR products by electroporation. The obtained colonies were cultured at 30°C for 20 h and purified using QIAGEN Plasmid Mini Kit (Qiagen). The sequences of the constructed sub-module-DNAs and RCR module-genomes were verified using iSeq 100 (Illumina). Detailed information on the constructed DNAs is provided in Supplementary Table 4, and the primers and assembly methods used are listed in Supplementary Table 5.

### Agarose gel electrophoresis and imaging

After all RCR reactions, replication intermediates were processed for finalization, allowing incomplete products to complete replication. After RCR reactions for DNA constructs (*in vitro* cloning), amplified products were subjected to five-fold dilution with 1 × RCR buffer I and II and incubated at 37°C for 15 min. After PURE-RCR reactions, amplified products were subjected to five-fold dilution with 1 × RCR buffer I and II supplemented with SCR enzyme mix [28] and 0.2 mg/ml RNaseA (QIAGEN, Cat no. 19 101), followed by incubation at 37°C for 15 min. Unless otherwise specified, 0.5 µl of the finalized product was loaded onto the agarose gel.

**Figure 3.**
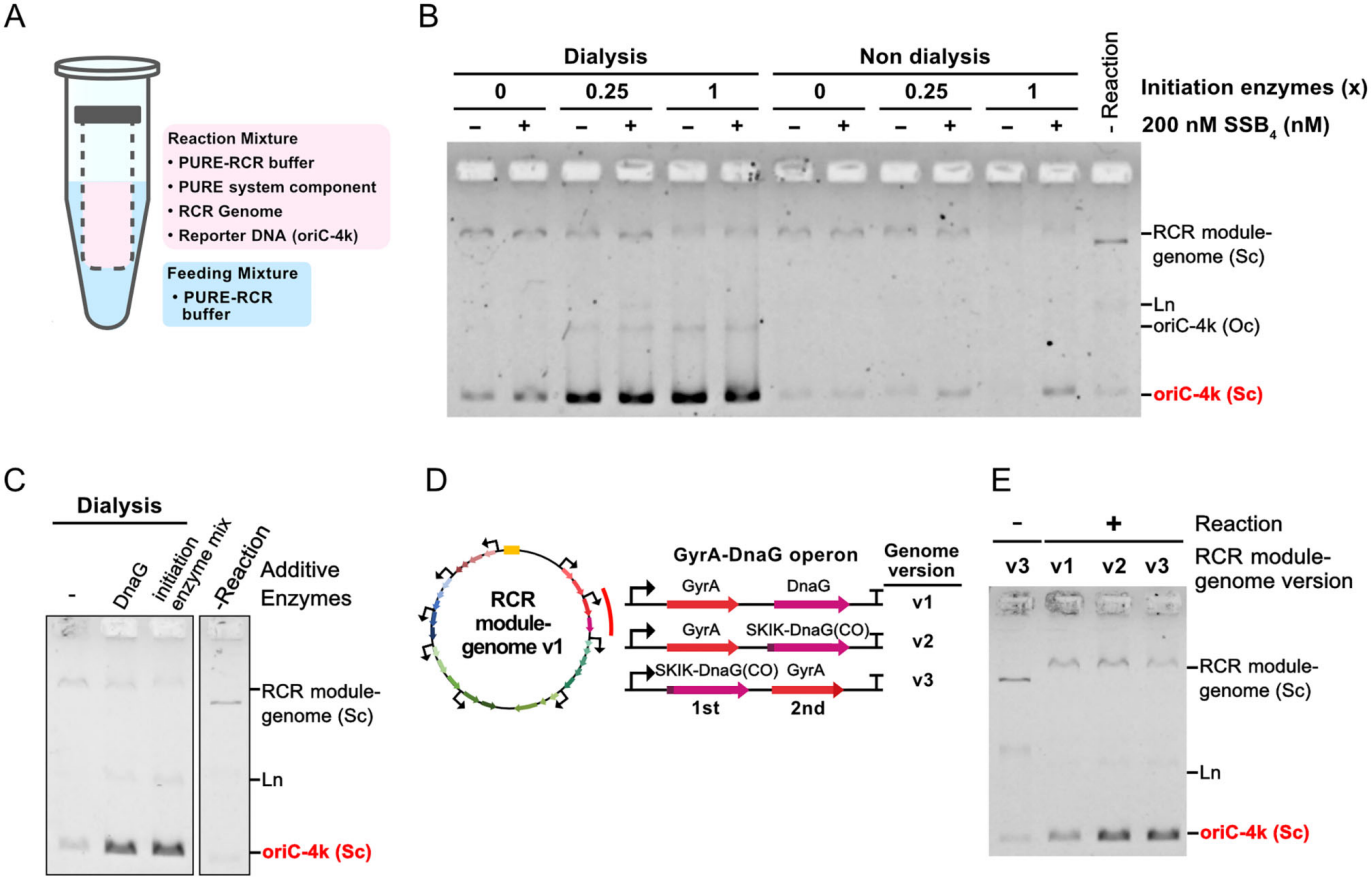
Improvement of RCR module-genome function via a dialyzed PURE-RCR system and genetic modification. (**A**) Schematic of the Dialyzed PURE-RCR system, which enhances protein expression by supplying substrates across a dialysis membrane. (**B**) Agarose gel electrophoresis showing enhanced DNA amplification in the dialysis system. Using 100 pM RCR module-genome and 250 pM reporter DNA (oriC-4k) as templates, the reaction was incubated for 9 h at 37°C, and products were separated on a 1% agarose gel (60 V, 60 min) and stained with SYBR Green I. Dialysis enabled faint amplification of the reporter DNA with only the RCR module-genome products. Amplification was further enhanced by the addition of a purified initiation enzyme mixture. (**C**) Identification of the rate-limiting factor within the initiation enzyme group. The addition of purified DnaG (25 nM) restored amplification to a level comparable to that with the initiation enzyme mixture (0.25× concentration), suggesting that DnaG is a rate-limiting factor. (**D**) Schematic of genetic modifications to enhance DnaG expression, including the addition of a SKIK tag and codon optimization (v2), and rearrangement of the gene within its operon (v3). (**E**) Electrophoretic analysis of reporter DNA amplification using the modified RCR module-genomes (v2, v3) after a 9-h incubation at 37°C.

**Figure 4.**
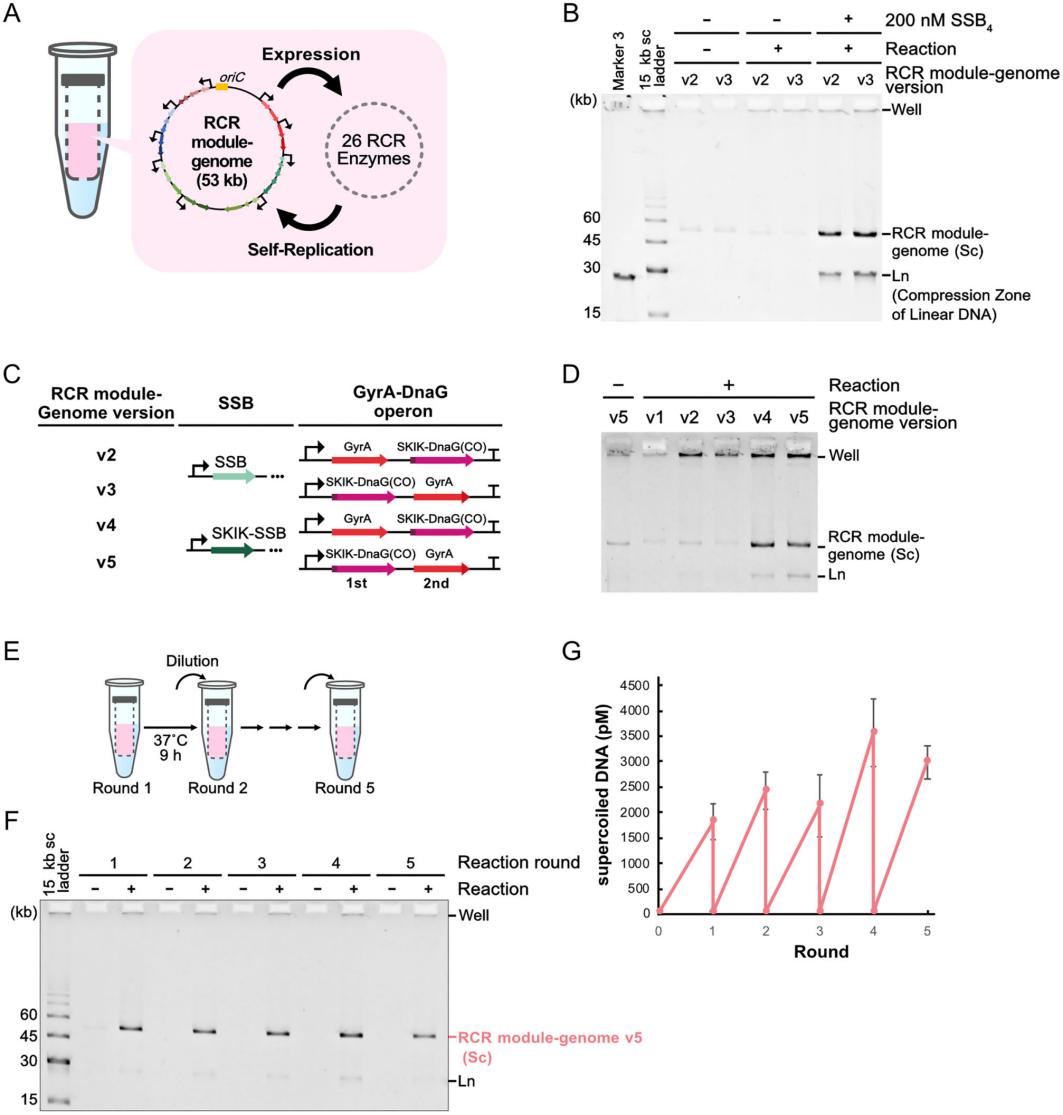
Achievement of recursive self-replication of the RCR module-genome. (**A**) Schematic of the self-replication assay. The reaction was performed with only the RCR module-genome as a template, excluding the reporter DNA (9 h, 37°C). (**B**) The effect of SSB on RCR module-genome self-replication. Using 100 pM RCR module-genome as a template, supercoiled genome amplification required purified SSB (200 nM). (**C**) Schematic of genetic modifications to enhance SSB expression by adding a SKIK tag, creating RCR module-genomes v4 and v5. (**D**) Self-replication of RCR module-genomes v4 and v5 containing SKIK-SSB. Using 100 pM of each genome as a template, self-replication occurred without any purified replication proteins. (**E**) Schematic of the serial transfer experiment. The amplified product was diluted to 50 pM and added to a fresh reaction mixture for the next reaction round. **(F)** Results of the serial transfer experiment. Starting from the 50 pM RCR module-genome v5, amplification was sustained up to reaction round 5. **(G)** Quantification of RCR module-genome v5 amplification during the serial transfer experiment. Amplified DNA was quantified from the supercoiled (sc) band intensity. Data are presented as mean ± standard deviation (SD; *n* = 3). All agarose gel images in this figure **(B, D, F)** show reaction products separated on a 0.5% agarose gel (60 V, 60 min) and stained with SYBR Green I. The leftmost lane contains a DNA size marker (15 kb supercoil ladder). Labels on the right indicate supercoiled (sc) and linear (ln) forms of the RCR module-genome. Source data is provided as a Source Data file.

Electrophoresis was performed using finalized products in a 0.5 × Tris-Borate-EDTA (TBE) buffer. Agarose gel concentrations of 1% (Figs 1–3) or 0.5% (Fig. 4) were used depending on the experiment, and detailed electrophoresis conditions are described in each Figure Legend. Gels were stained with SYBR Green I and imaged using FUSION SOLO.7S.EDGE (Vilber). Band intensity was analyzed using ImageJ software [33]. Mk3 (Nippon Gene) and 15 kb supercoil ladder were used as DNA markers.

The 15 kb supercoil ladder marker was prepared by utilizing the multimerization property of *E. coli* ME9783. *Escherichia coli* ME9783 cells were transformed with a 15-kb plasmid to generate multimeric plasmid forms, and plasmid DNA was then extracted to obtain a supercoiled ladder [34].

### Rescue assay of each RCR enzyme

Rescue assays were performed to evaluate whether proteins expressed in the PURE system could functionally restore RCR reactions lacking specific replication factors. First, target proteins were expressed using PUREfrex 2.0 (GeneFrontier) according to the manufacturer’s protocol, with pET plasmids (containing T7 promoter/terminator sequences) as templates. Details of the templates are provided in Supplementary Table 1. The expression reaction was performed in a 10 µl volume at 33°C for 3 h.

The PURE reaction containing the expressed proteins was then added to RCR reactions lacking specific replication factors. The PURE system products were added at a final concentration of 10%. For the supplementary enzymes rescue assay, PURE-expressed supplementary enzymes were individually added at final concentrations of 1%, 3%, and 10%. RCR reactions were performed at 37°C for 10 h using oriC-16k as a reporter plasmid. RCR enzymes other than the tested replication factor were added at previously described concentrations [27].

### PURE-RCR reporter assay

Reactions contained RCR module-genome (100 pM), reporter plasmid oriC-4k (250 pM), and the T5 buffer composition described in Supplementary Table 6. This T5 buffer was a custom buffer optimized for PURE-RCR reactions and was used consistently in all subsequent PURE-RCR experiments.

The PURE-RCR reaction system includes the PURE system supplemented with chaperones, RNase inhibitor, and exonuclease mixture. This PURE-RCR reaction mixture was used consistently in all subsequent PURE-RCR experiments. PUREfrex custom (GeneFrontier) was used as the PURE system. Solution II (∆T7 RNAP) was added at 1 × concentration and Solution III at 1000 nM. T7 RNA polymerase from GeneFrontier (7.5 nM; Figs 2–4) was used. RNase Inhibitor Murine (NEB, M0314S) was added at 0.8 U/µl, and molecular chaperones DnaK mix (GeneFrontier, PF003) at 0.5 × and GroE mix (GeneFrontier, PF004) at 1 × were included. Exonuclease I (200 mU/µl, NEB) and Exonuclease V (RecBCD, 50 mU/µl, NEB) were added as an exonuclease cocktail. RecG 60 nM, RecJ 0.5 U/μl, ExoIII 60 mU/μl were added following previous experiments [32]. This exonuclease cocktail, RecG and RecJ were included to suppress replication intermediates during the amplification of large DNA templates. Reactions were performed in a 10 µl volume at 37°C for 9 h. In some experiments, purified replication proteins were supplemented at concentrations defined as 1 × based on previously reported values [27].

### Dialyzed PURE–RCR reporter assay

To enhance expression levels through continuous substrate supply, dialyzed PURE–RCR reactions were performed using a dialysis-based PURE system as described previously [11]. The dialysis buffer consisted of T5 buffer (Supplementary Table 6) without tRNA, supplemented with 50 µg/ml carbenicillin as previously described [11]. A volume of 150 µl dialysis buffer was added to a 2 ml tube. An Xpress Micro Dialyzer (Scienova, MD100, 10–100 µl, molecular weight cut-off (MWCO) 6–8 kDa) containing 10 µl reaction mixture was placed into the 2 ml tube containing the dialysis buffer, and the reaction was performed with dialysis. Reactions were performed at 500 revolutions per minute and 37°C for 9 h. In some experiments, purified replication proteins were supplemented at concentrations defined as 1 × based on previously reported values [27].

### Dialyzed PURE–RCR self-replication assay

To evaluate the self-replication capacity of the RCR module-genome, dialyzed PURE-RCR was performed with only the RCR module-genome as the DNA template. Reactions were carried out in a 10 µl volume with RCR module-genome (100 pM) as template, and Dialyzed PURE–RCR reactions were performed at 37°C for 9 h. In some experiments, purified replication proteins were added.

### Serial transfer experiment

To assess continuous self-replication capacity of the RCR module-genome, serial transfer experiments were performed. Dialyzed PURE-RCR reactions were initiated at an initial concentration of 50 pM. After each reaction round, the supercoiled product concentration was quantified by band intensity (see below), and an aliquot of the unpurified reaction mixture was directly diluted with fresh reaction mixture so that the input concentration of the next reaction round was again 50 pM. Because the unpurified reaction was used as the seed for the subsequent reaction round, the dilution fold applied at each transfer was numerically equivalent to the fold amplification of the supercoiled product in that reaction round (typically 28- to 86-fold; Supplementary Tables 7 and 8). Serial transfer was carried out for five reaction rounds. To suppress nonspecific amplification, Dextran 200 000 (Fujifilm, 041-22 612, Lot. CAP5186) was added as a molecular crowding agent at a concentration of 8% (w/v) to both the reaction mixture (inner solution) and the dialysis buffer (outer solution) in each reaction round.

Quantification was performed by loading serially diluted DNA samples (two-fold dilution series) on the same agarose gel and analyzing sc DNA band intensity using ImageJ. 0.07 µl of the finalized product was loaded onto the agarose gel. Three independent experiments (*n* = 3) were performed, and results are shown as mean ± SD. The total number of doublings was calculated from the amplification fold. The amplified DNA products were subjected to sequencing using an iSeq 100 system. Sequencing libraries were prepared using the NEBNext Ultra II FS DNA Library Prep Kit with Multiplex Oligos for Illumina (NEB). Sequence errors were analyzed using Geneious Prime 2026.0.2.

### DNA size verification by restriction-enzyme digestion

The reporter plasmid was digested with ScaI. The RCR module-genome was digested with NotI and BsaI. Reactions were performed in CutSmart Buffer (NEB) at a final enzyme concentration of 0.5 U/μl and incubated at 37°C for 1 h. The products were detected by agarose gel electrophoresis.

### Sodium dodecyl sulphate–polyacrylamide gel electrophoresis analysis

*in vitro* translation products were detected by adding fluorescently labeled lysyl–tRNA (0.5 μl, FluoroTect GreenLys, Promega) to a 10 μl PUREfrex reaction. Samples were then heated at 95°C for 1 min and resolved on a 16.5% polyacrylamide gel (p-PAGEL, ATTO) at 200 V for 75 min. The gel was first imaged for in-gel fluorescence and subsequently stained with Coomassie Brilliant Blue (CBB).

### Statistical analyses

Quantitative data are presented as the mean ± SD from three independent experiments (*n* = 3), as indicated in the figure legends and source data. The value of *n* represents independent experimental replicates.

## Results

### RCR proteins expressed in PURE system are active

To verify the functional activity of each of the 26 RCR proteins expressed in the PURE system, we performed a series of rescue assays (Fig. 1B). A protein or protein complex of interest (POI) was expressed in the PURE system using a pET vector containing T7 promoter and T7 terminator. An aliquot of each PURE reaction was then added to an RCR mixture lacking the POI. Rescue of RCR activity was monitored by amplification of a 16-kb reporter plasmid containing *oriC* and two inward-facing *terB* sites (oriC-16k) (Fig. 1B and Supplementary Fig. S1). This arrangement blocks the re-entry of replication forks into *oriC* through the Tus–*ter* fork-trap system, thereby suppressing rolling-circle replication [31]. Using this rescue assay, we examined the 26 RCR proteins by dividing them into 8 initiation enzymes (DnaA, IhfA, IhfB, DnaB, DnaC, DnaG, GyrA, and GyrB), 13 elongation enzymes (single-stranded DNA-binding protein (SSB), PolA, LigA, TopB, DnaX, HolA, HolB, HolC, HolD, DnaE, DnaQ, HolE, and DnaN), and 5 supplementary enzymes (RecQ, RnhA, ParC, ParE, and Tus).

We first tested the initiation enzymes group. DnaA, DnaG, GyrA, and GyrB were expressed individually. IhfA/IhfB and DnaB/DnaC were co-expressed as operons. In a control experiment that included all purified RCR proteins, amplification of supercoiled reporter plasmid was observed, accompanied by concatemer, linear, and nicked DNA byproducts (Fig. 1C). Concatemer DNA is produced due to rolling-circle replication [27]. Linear and nicked DNA are produced likely due to DNA damage during RCR. All PURE-expressed initiation enzymes rescued the RCR amplification of the supercoiled reporter plasmid (Fig. 1C). As expected, in the absence of an expression plasmid in the PURE reaction, no amplification was observed. Notably, faint amplification was detected in the absence of IhfAB, indicating that they play a stimulatory, but not essential, role in RCR.

Next, we tested the elongation enzymes group. For the rescue assays, SSB, DnaN, PolA, LigA, and TopB were expressed individually. The eight subunits of the Pol III* complexes (DnaX, HolA, HolB, HolC, HolD, DnaE, DnaQ, HolE) were co-expressed from two operons on a single vector. All PURE-expressed products rescued RCR amplification of the supercoiled reporter DNA (Fig. 1D). The assays for PolA, SSB, and TopB also produced DNA bands smaller than the reporter plasmid, likely due to DNA cleavage and re-ligation caused by insufficient protein levels. Furthermore, in the assay lacking TopB, a moderate amplification as concatemer DNA was detected, probably due to rolling circle amplification caused by incomplete decatenation. This observation is consistent with a previous report that using only Topo IV for termination in the RCR system results in the formation of concatemers [27].

In the rescue assay for the supplementary enzymes group, amplification of a supercoil, concatemer and nicked DNA was observed even in reactions lacking all of the supplementary enzymes, indicating they are not essential for DNA amplification (Fig. 1E). With the addition of the supplementary enzymes mixture, concatemer and open-circular byproducts were reduced and supercoiled monomers were amplified as a single major band. To identify the factors responsible for this effect, we performed rescue assays by individually adding each purified or PURE-expressed protein to the reaction lacking supplementary enzymes. Purified RNase H (RnhA) reduced open-circular DNA, likely due to resolving R-loops. We also observed the same effect with PURE-expressed proteins. Purified Tus reduced concatemers, consistent with previous reports [31]. These experiments confirmed that PURE-expressed RNase H and Tus are functionally active. The addition of purified RecQ or ParC/E (Topo IV) had no significant effect on this assay. Although RecQ is a helicase that stimulates Topo III–mediated decatenation, Topo III alone can drive sufficient decatenation [27]. Although Topo IV can also catalyze decatenation, the decatenation activity of Topo III alone was sufficient for this assay. In summary, we successfully substituted the 23 proteins essential for the RCR amplification of supercoiled DNA with their PURE system-expressed products. Although we could not fully confirm the activity of the expressed RecQ and ParC/E, we decided to include them in subsequent experiments, considering their potential supportive roles in the RCR.

### Construction of RCR module-genome encoding all 26 RCR proteins

A key challenge in building self-replicating systems is to design and construct a large, stable genome that encodes all the necessary components. To address this challenge, we designed and constructed the RCR module-genome, a single 53 kb circular DNA molecule encoding all 26 RCR genes (Fig. 2A). To ensure stability, our design mimics the organization of natural genomes. Transcription occurring on the replicating DNA template can collide with replication forks. In the co–directional configuration, replication inhibition due to collisions is limited, whereas head–on encounters cause fork slowing or stalling and R–loop-dependent blockage [35–37]. To minimize this effect of transcription, the transcription direction of each gene was aligned with the progression direction of the replication forks, which proceed bidirectionally from the origin of replication (*oriC*). To minimize the number of promoters, we designed operons containing 2 to 5 genes, each kept below approximately 5 kb (Supplementary Table 2). In general, genes encoding proteins that form functional complexes were placed within the same operon, including the DnaB-DnaC complex, IHF complex (IhfA and IhfB), Topo IV complex (ParC and ParE), Pol III core complex (DnaQ, HolE, and DnaE), and clamp-loader complex (HolC, HolD, HolB, HolA, and DnaX). Each operon was transcribed by a T7 promoter. Additionally, highly efficient T7hyb10 terminators were placed downstream of each operon [30]. Most genes carry an optimized RBS derived from the 5′ untranslated region of the pET21a vector, with exceptions listed in Supplementary Table 9.

As the first step of RCR module-genome construction, each operon was assembled by multi-fragment assembly into a vector containing a T7 promoter and T7hyb10 terminator, yielding nine operon plasmids (Fig. 2A). In the second step, three sub-module-DNAs were constructed by assembling PCR-amplified operon fragments with an oriC-vector via Assembly-RCR cloning. During the assembly step, DNA fragments were assembled via homologous ends, and the subsequent RCR selectively amplified only circularized products containing *oriC*. This procedure yielded a 22-kb initiation enzymes sub-module-DNA composed of three operons, a 30-kb elongation enzymes sub-module-DNA composed of four operons, and a 17-kb supplementary enzymes sub-module-DNA composed of two operons. Then, the oriC-vector region (8 kb) was removed from the elongation enzymes and supplementary enzymes sub-module-DNAs by AvrII digestion. Finally, these digested fragments, along with the oriC-containing initiation enzymes fragment similarly digested with AvrII, were then assembled by pre-designed homologous sequence and amplified via Assembly-RCR to obtain the complete 53 kb RCR module-genome. Restriction enzyme analysis confirmed the 53 kb DNA structure (Supplementary Fig. S2).

### Functional assessment of RCR module-genome in PURE-RCR system

To assess the activity of proteins expressed from the RCR module-genome, we established a one-pot PURE-RCR system where transcription-translation and DNA amplification occur simultaneously. The system was supplemented with an RNase inhibitor and chaperone mixtures (DnaK and GroE mixes) to enhance protein expression and activity [29]. Furthermore, to suppress replication intermediates during the amplification of large DNA, we added a cocktail of exonucleases ExoI, ExoV, RecG, RecJ, and ExoIII [32, 38]. Initial attempts to amplify the RCR module-genome were unsuccessful, likely due to its large size and multiple promoter sites. Therefore, we employed a reporter assay for a more sensitive assessment of its activity (Fig. 2B). This assay uses a small, T7 promoter-less reporter plasmid (oriC-4k) as the amplification template.

Even using the reporter assay, proteins expressed from the RCR module-genome alone failed to amplify the reporter DNA, suggesting the expression of one or more proteins was insufficient. To identify the limiting factors, we tested combinations of purified proteins in the reporter assay. We added SSB, which is required in high abundance, and mixtures of the functional protein groups. Reporter amplification was observed only when both SSB and the initiation enzymes mixture were added (Fig. 2C). This result indicates that SSB and some proteins of initiation enzymes components (DnaA, DnaG, GyrA, GyrB, IhfA, IhfB, DnaB, DnaC) are limiting factors. The RCR module-genome itself did not amplify under this condition. When full components of purified RCR proteins were supplemented, both the RCR module-genome and the reporter DNA were amplified.

### Improvement of RCR module-genome function through dialyzed PURE-RCR system and genetic modification

Since DNA amplification driven solely by proteins expressed from the RCR module-genome was undetectable, we adopted a strategy to increase protein expression levels. In the PURE system, substrates such as amino acids, nucleotides, and ATP are consumed during transcription and translation, causing the reaction to stall as they are depleted. To overcome this limitation, we applied a method of dialyzed PURE system in which the reaction mixture is enclosed within a semipermeable membrane, allowing continuous diffusion of small-molecule substrates from an outer reservoir while retaining macromolecules such as proteins and DNA [11, 39]. The dialyzed method was therefore used for subsequent PURE-RCR experiments (Fig. 3A; see the ‘Materials and methods’ section for details).

The Dialyzed PURE-RCR system allows a modest level of reporter DNA amplification driven solely by proteins expressed from the RCR module-genome (Fig. 3B). Although the addition of SSB had no effect on this amplification, supplementing the initiation enzymes mixture was still required for a higher level of the reporter amplification. This level of the reporter amplification in the dialyzed system was significantly higher than that in the nondialyzed system containing SSB and the initiation enzymes mixture. Suspecting that DnaG was the next bottleneck, due to its crucial role in lagging-strand synthesis alongside SSB, we tested its effect directly. The addition of purified DnaG alone was sufficient to rescue reporter amplification to a level seen with the full initiation enzymes mixture (Fig. 3C). These results pinpointed DnaG as the primary rate-limiting factor in the system.

To address the insufficient expression of DnaG, we modified the DnaG gene encoded in the RCR module-genome. Specifically, we introduced SKIK-DnaG codon-optimized (CO), which combines an expression-enhancing SKIK tag with codon optimization (Fig. 3D, v2). The SKIK tag encodes the four amino acids Ser–Lys–Ile–Lys immediately downstream of the initiating methionine and is known to increase translation and relieve downstream translational arrest [40, 41]. It is also known that wild–type *E. coli* DnaG contains a high frequency of rare codons [42]. Consistent with this, the RCR module-genome v2 harboring SKIK-DnaG (CO) showed higher DnaG expression (Supplementary Fig. S3) and also increased reporter DNA amplification (Fig. 3E). We further examined RCR module-genome v3 (Fig. 3D, v3), in which the position of the SKIK-DnaG (CO) gene in the GyrA-DnaG operon was rearranged to the beginning of the operon [43], resulting in no significant enhancement of the reporter DNA amplification (Fig. 3E, v2 and v3). Thus, by combining the dialyzed system with a genetically enhanced RCR module-genome, we generated an active RCR system solely from the expression products of the RCR module-genome.

### Achieving recursive self-replication of the RCR module-genome

In the reporter assay described above, amplification of the RCR module-genome itself was not observed (Fig. 3E). To demonstrate self-replication, in which proteins expressed from the RCR module-genome amplify the DNA itself, we next performed the dialyzed PURE-RCR reaction without the reporter DNA (Fig. 4A). Even when the improved RCR module-genomes (v2 and v3) were assessed, no amplification of supercoiled DNA was detected (Fig. 4B, −SSB, +Reaction). When purified SSB was supplied, supercoiled amplification of the RCR module-genome along with the linear form was observed with a similar level between v2 and v3 (Fig. 4B, +SSB).

This result identified SSB as the next critical limiting factor for self-replication. To increase the SSB expression, we constructed RCR module-genome v4 and v5 by introducing an SKIK tag into the SSB gene in the RCR module-genome v2 and v3, respectively (Fig. 4C). Consequently, the modified RCR module-genomes v4 and v5 carrying SKIK-SSB showed higher SSB expression (Supplementary Fig. S4) and allowed amplification of the RCR module-genome itself as a supercoiled form along with replication intermediates seen in the gel-well and a slight amount of the linear form (Fig. 4D). This result marked the achievement of RCR module-genome self-replication, a system we termed PRIMES.

We further confirmed expression of the RCR proteins from the RCR module-genome by sodium dodecyl sulphate–polyacrylamide gel electrophoresis. Bands corresponding to a substantial fraction of the 26 proteins were detected at their expected molecular weights (Supplementary Fig. S5). Because a few bands were difficult to assign on this gel, we additionally examined expression from each operon fragment of the RCR module-genome, which allowed nearly all 26 proteins to be detected (Supplementary Fig. S6).

To demonstrate recursive self-replication, we performed serial transfers of the replication products into fresh reaction mixtures to test if the reaction could be serially propagated. We initiated the first reaction round with 50 pM of the RCR module-genome and subsequently diluted the product to the same concentration for each new reaction round (Fig. 4E). A challenge in this process was the generation of smaller DNA byproducts other than the target RCR module-genome (Supplementary Fig. S7). To suppress this, we adopted a previously described method, supplementing the reaction with the molecular crowding agent Dextran [28]. Dextran selectively suppressed the generation of smaller DNA byproducts that otherwise occurred in later reaction rounds (Supplementary Fig. S7).

In the serial transfer experiment using the RCR module-genome v5, the first reaction round of self-replication yielded a 36-fold amplification of the RCR module-genome as supercoiled DNA (1816 ± 355 pM) (Fig. 4F and G). After dilution to 50 pM, this product seeded a second reaction round that resulted in a 49-fold amplification (2427 ± 363 pM). Recursive self-replication was sustained over three additional reaction rounds, with mean fold amplifications of 43-, 71-, and 60-fold in rounds 3, 4, and 5, respectively (Fig. 4G and Supplementary Table 7). In total, five reaction rounds yielded approximately 28 doublings (2²⁸-fold cumulative amplification) of the RCR module-genome. Similarly, 26 doublings of self-replication were observed using the RCR module-genome v4 (Supplementary Fig. S8 and Supplementary Table 8). These results demonstrate that the RCR proteins expressed from the RCR module-genome can stably drive its recursive self-replication. It should be noted that NGS analysis of the final serial-transfer products of RCR module-genome v5 identified major mutations at only two positions within *oriC* (Supplementary Fig. S9). One of them was an evolved-type mutation already reported [44].

## Discussion

In this study, we constructed a 53 kb circular DNA, the RCR module-genome, encoding all 26 proteins of the reconstituted *E. coli* replication cycle (RCR), and established the PRIMES system in which the DNA replicates itself via expression by the PURE system. This is the first report of a PURE–RCR system where RCR proteins expressed in the PURE system amplify their own template DNA. The PURE–RCR platform reconstitutes a genome replication module that couples transcription/translation (PURE) with DNA replication (RCR). Increasing gene expression through gene modifications was critical to achieving self-replication. We further showed that the system can be sustained by serial transfer, in which products are diluted and added to fresh reactions. We successfully reconstituted a recursive DNA self-replication system, representing a critical milestone toward the construction of artificial cells and establishing a foundational module for genome self-replication.

We have established self-replication of the genome-replication module, in which the RCR module-genome encodes all 26 replication proteins and generates them to replicate its own DNA. Toward cellular-level self-reproduction, additional functional modules will need to be encoded and integrated. The present system still relies on the externally supplied PURE system for transcription and translation, as well as biochemical substrates such as amino acids, nucleotides, and energy sources that are replenished through dialysis. Recent progress in reconstructing genetically encoded transcription–translation components, including aminoacyl–tRNA synthetases, tRNAs, translation factors, and ribosomal components, suggests a route toward a self-regenerating expression module [7, 9, 10, 12–18]. Because RCR can amplify circular DNA up to 2 Mb [28], progressive integration of these functional modules into the RCR module-genome should be feasible and will pave the way toward a self-reproducing artificial cell genome.

We established the self-replication system by iteratively identifying bottlenecks and resolving them through rational design. The defined, reconstituted nature of the system, comprising 26 purified proteins, enabled the systematic identification of bottlenecks via selective complementation assays. This approach identified SSB and DnaG as the key rate-limiting factors. Both proteins are critical for lagging-strand synthesis. This bottleneck can be explained by a cascade where insufficient DnaG primase activity reduces priming frequency. This generates longer Okazaki fragments and, consequently, increases the demand for SSB to coat the exposed single-stranded DNA [45]. In turn, a deficit in SSB inhibits replication fork progression and suppresses DnaG priming, leading to a complete stall of the reaction [46]. Based on this insight, we rationally engineered the RCR module-genome to enhance the expression of both proteins using codon optimization and expression-enhancing tags, which successfully enabled self-replication.

We demonstrated sustained self-replication over five reaction rounds. In initial trials without Dextran, smaller DNA byproducts appeared after reaction round 2 (Supplementary Fig. S7). The emergence of such “parasitic” DNA is often unavoidable, particularly in open bulk reactions [47, 48]. The amplification of a large 53 kb DNA template increases the risk of double-strand breaks (DSBs) during RCR amplification. Such truncated fragments can be re-circularized by DNA Ligase in the RCR system, generating smaller circular DNA that is amplified more rapidly than the full length RCR module-genome. We here found that Dextran supplementation efficiently suppressed the formation of these smaller DNA byproducts (Supplementary Fig. S7), likely by stabilizing DNA and/or protein function and thereby reducing DSB-mediated DNA damage. Although we tested only five reaction rounds in this study, Dextran supplementation enabled sustained and stable serial self-replication of the RCR module-genome.

Through the development of the PRIMES system, two major challenges became apparent, as described above: insufficient gene expression levels and the emergence of parasitic DNA. These issues are likely to re-emerge in the future when constructing larger genomes or when carrying out more continuous serial passaging. In particular, as the number of genes increases in the larger genome, expression resources for individual genes are expected to become limiting, making optimization of gene expression increasingly important. Addressing these challenges will be a key step toward improving the robustness of the PRIMES system.

Previously reported recursive self-replication systems have utilized phi29 phage replication and have demonstrated self-replication of DNA templates of 2.7 or 5.9 kb, encoding a small number of components [20,21,49]. By contrast, our work represents a major advance in both complexity and scale, reconstituting the *E. coli* chromosomal replication machinery to self-replicate a DNA longer than 50 kb. Because RCR can amplify DNA up to 2 Mb [28], the 53 kb RCR module-genome can be progressively expanded to incorporate genes responsible for transcription, translation, and other essential processes, providing a platform for the construction of artificial cell genomes.

Adaptive evolution of the *oriC* sequence has been reported using RCR [44]. In addition, directed evolution of individual proteins has been achieved using other self-replication systems [22,49,50]. Whereas these studies were limited to evolving a single *cis-*acting DNA element or individual proteins, our PRIMES system opens the door to system-level evolution, enabling optimization of expression levels across all 26 genes and adaptation of the RCR machinery to specific *in vitro* environments.

## Supplementary Material

gkag663_Supplemental_Files

## Data Availability

All DNA sequences used in this study, including plasmids for rescue assays, operon plasmids, sub-module-DNAs, and RCR module-genomes, are provided in Supplementary Tables 1–5 and in GenBank format as Supplementary Data. All other data supporting the findings of this study are available within the paper and its supplementary information files. Source data for figures are provided with this paper.
