# Supplementary material for: A self-replicating artificial module-genome that generates bacterial chromosome replication system in vitro
Source: Nucleic Acids Res. 2026 Jul 6;54(13):gkag663. doi: 10.1093/nar/gkag663 (PMC13333201; doi:10.1093/nar/gkag663)

Supplementary Information for

A self-replicating artificial module-genome that generates bacterial chromosome replication system *in vitro*

Yamagishi *et al.,*

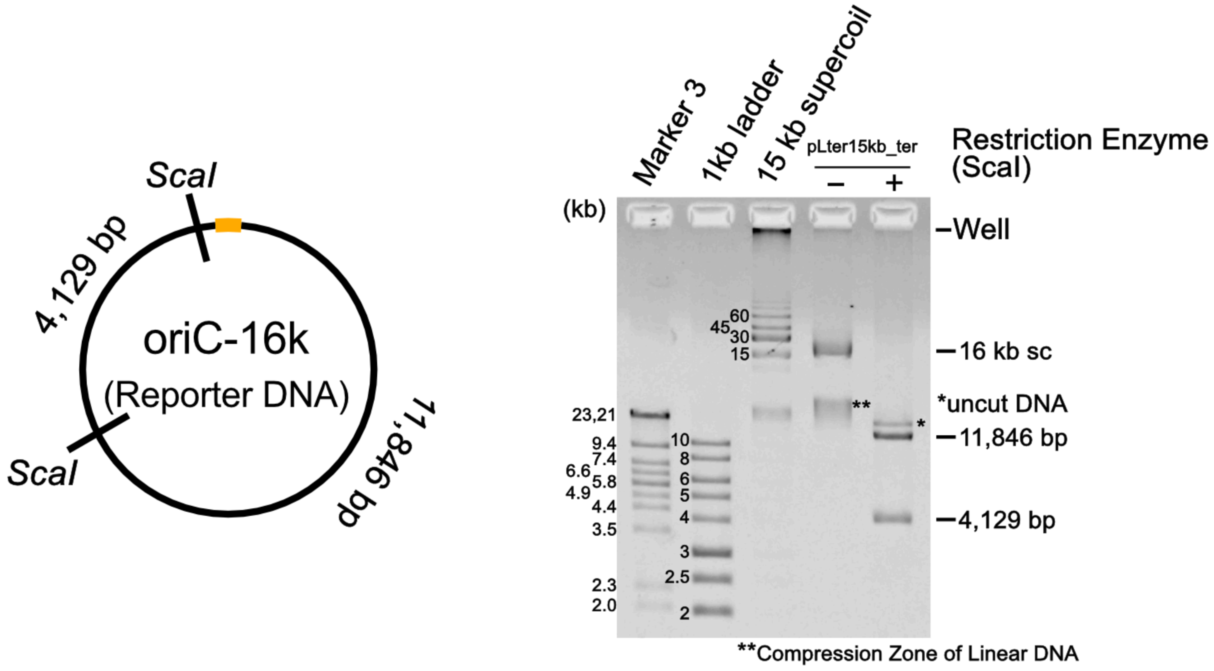

Supplementary Figure 1 Restriction-enzyme verification of the oriC-16k reporter DNA. The left panel shows the map of the oriC-16k reporter DNA with the two ScaI sites. The right panel shows agarose gel electrophoresis of the reporter DNA before and after ScaI digestion. ScaI digestion produced the expected fragments of 11846 bp and 4129 bp. The undigested sample contained supercoiled DNA. The double asterisk indicates the compression zone of linear DNA.

Alt text: Circular map of the oriC-16k reporter DNA and an agarose gel image showing ScaI digestion. The digestion produced the expected restriction fragments and verified the structure of the oriC-16k reporter DNA used in the rescue assay.

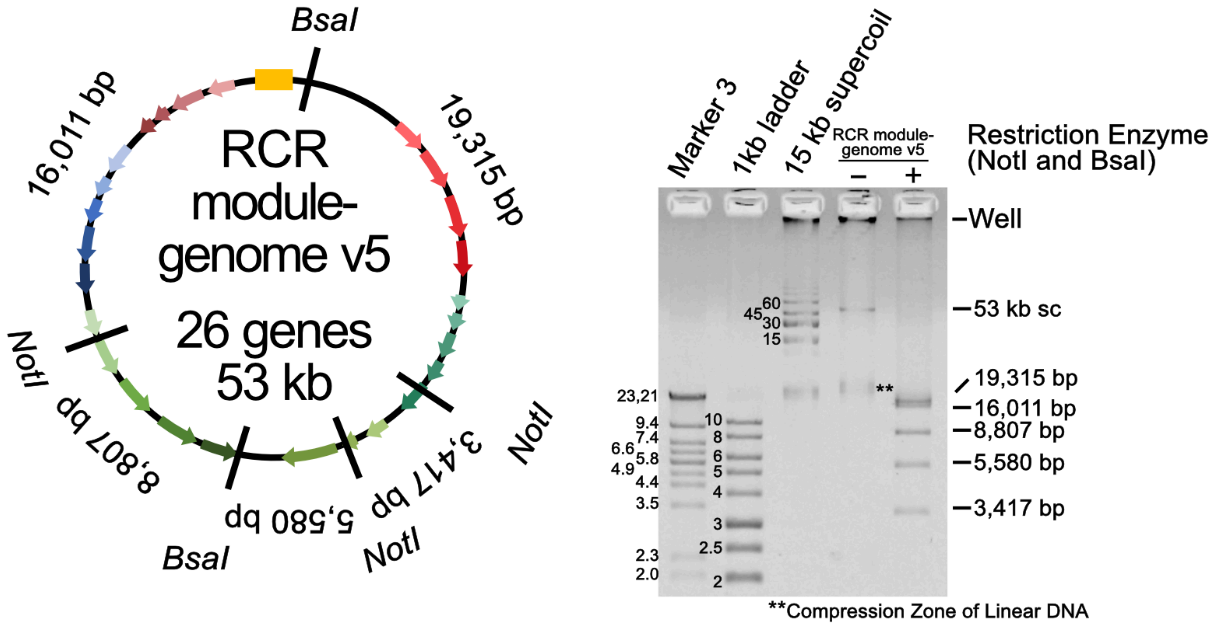

Supplementary Figure 2 Restriction-enzyme verification of the RCR module-genome v5. The left panel shows the map of the 53 kb RCR module-genome v5 and the NotI and BsaI restriction sites used for structural verification. The right panel shows agarose gel electrophoresis of the RCR module-genome before and after digestion with NotI and BsaI. The digested sample showed the expected restriction pattern for the 53 kb RCR module-genome. The double asterisk indicates the compression zone of linear DNA.

Alt text: Circular map of the 53 kb RCR module-genome v5 and an agarose gel image showing NotI and BsaI digestion. The digested sample showed the expected restriction pattern and verified the structure of the full-length RCR module-genome.

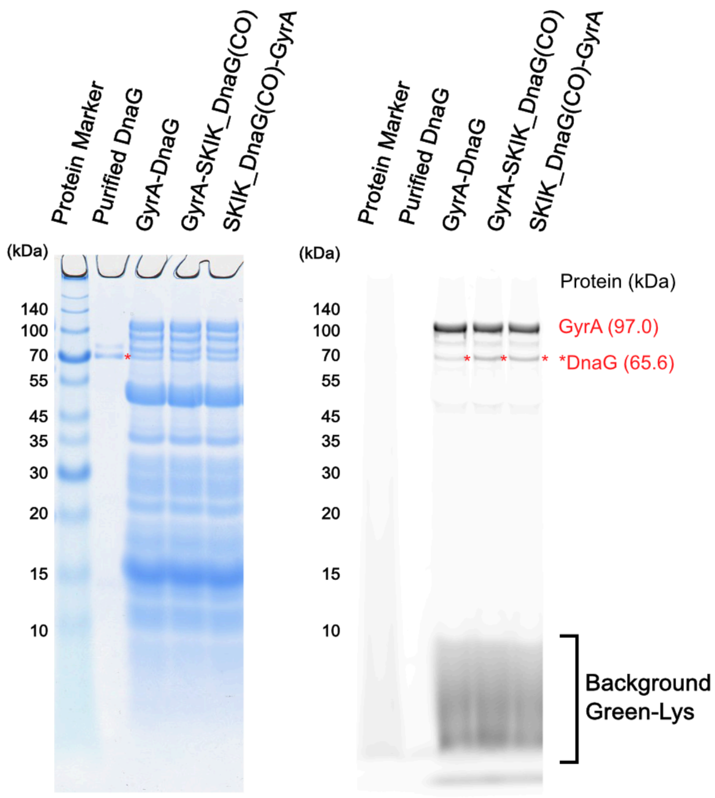

Supplementary Figure 3 Expression analysis of DnaG variants in the PURE system. GyrA-DnaG operon fragments encoding wild-type DnaG or SKIK-DnaG with codon optimization were expressed in the PURE system in the presence of FluoroTect GreenLys. Translation products were analyzed by SDS-PAGE followed by CBB staining and fluorescence imaging. Bands corresponding to GyrA and DnaG were detected at the expected molecular weights. DnaG bands are indicated by red asterisks. Introduction of the SKIK tag and codon optimization modestly increased the DnaG signal.

Alt text: SDS-PAGE analysis of DnaG expression from GyrA-DnaG operon variants in the PURE system. CBB staining and FluoroTect GreenLys fluorescence show that SKIK tagging and codon optimization modestly increased the DnaG band marked by red asterisks.

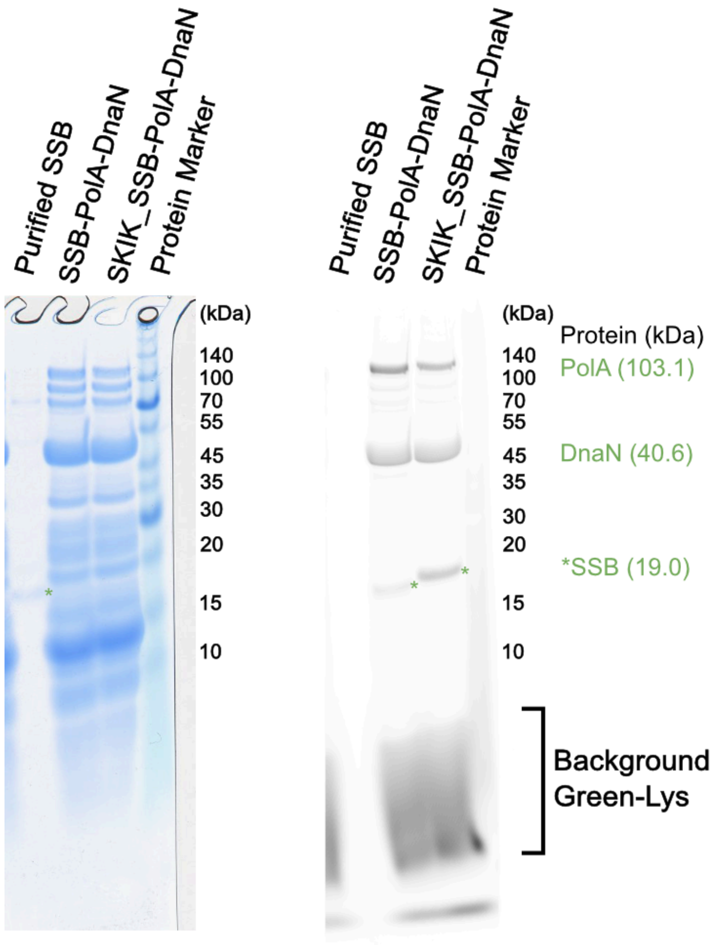

Supplementary Figure 4 Expression analysis of SSB variants in the PURE system. Operon fragments encoding wild-type SSB or SKIK-tagged SSB together with PolA and DnaN were expressed in the PURE system in the presence of FluoroTect GreenLys. Translation products were analyzed by SDS-PAGE followed by CBB staining and fluorescence imaging. Bands corresponding to PolA, DnaN and SSB were detected at the expected molecular weights. SSB bands are indicated by green asterisks. Introduction of the SKIK tag increased the SSB signal.

Alt text: SDS-PAGE analysis of SSB expression from SSB-PolA-DnaN operon variants in the PURE system. CBB staining and FluoroTect GreenLys fluorescence show increased SSB expression from the SKIK-tagged SSB construct marked by green asterisks.

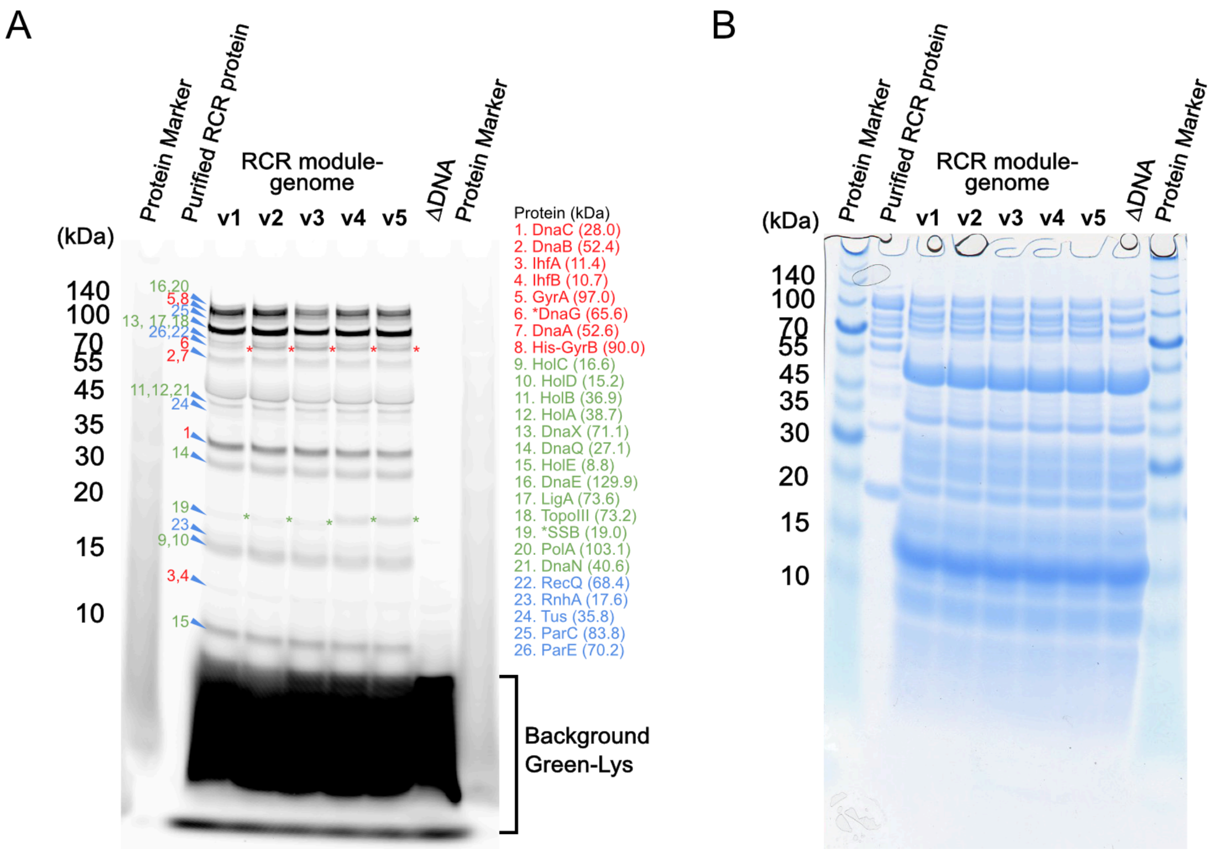

Supplementary Figure 5 Expression analysis of RCR proteins from the full-length RCR module-genome. RCR module-genome variants v1 to v5 were expressed in the PURE system in the presence of FluoroTect GreenLys. Translation products were analyzed by SDS-PAGE followed by fluorescence imaging (A) and CBB staining (B). Purified RCR proteins were loaded as molecular-weight references. Bands corresponding to many of the 26 RCR proteins were detected from the full-length RCR module-genome. Some proteins could not be assigned unambiguously because of overlapping bands. DnaG and SSB are indicated by red and green asterisks, respectively. The SKIK-tagged versions of DnaG and SSB increased the corresponding protein signals in the modified RCR module-genomes.

Alt text: SDS-PAGE analysis of proteins expressed from full-length RCR module-genome variants v1 to v5. FluoroTect GreenLys fluorescence and CBB staining show bands corresponding to many RCR proteins, with DnaG and SSB marked by red and green asterisks, respectively.

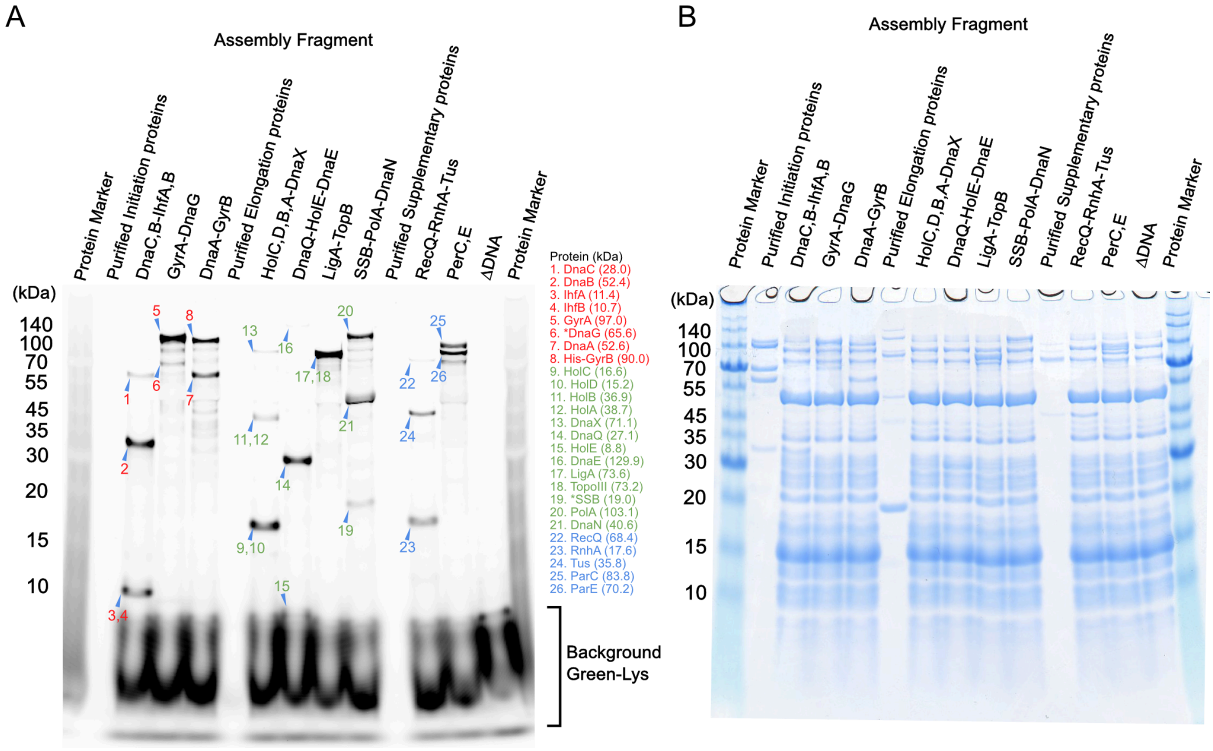

Supplementary Figure 6 Expression analysis of RCR proteins from individual operon fragments used for RCR module-genome assembly. Each assembly fragment was expressed in the PURE system in the presence of FluoroTect GreenLys. Translation products were analyzed by SDS-PAGE followed by fluorescence imaging (A) and CBB staining (B). Purified initiation proteins, elongation proteins and supplementary proteins were loaded as molecular-weight references. Expression of nearly all 26 RCR proteins was detected at the expected molecular weights. The analysis of individual operon fragments resolved several bands that were difficult to assign in the full-length RCR module-genome expression assay.

Alt text: SDS-PAGE analysis of proteins expressed from individual operon fragments used for RCR module-genome assembly. FluoroTect GreenLys fluorescence and CBB staining show expression of nearly all 26 RCR proteins, with purified protein mixtures included as molecular-weight references.

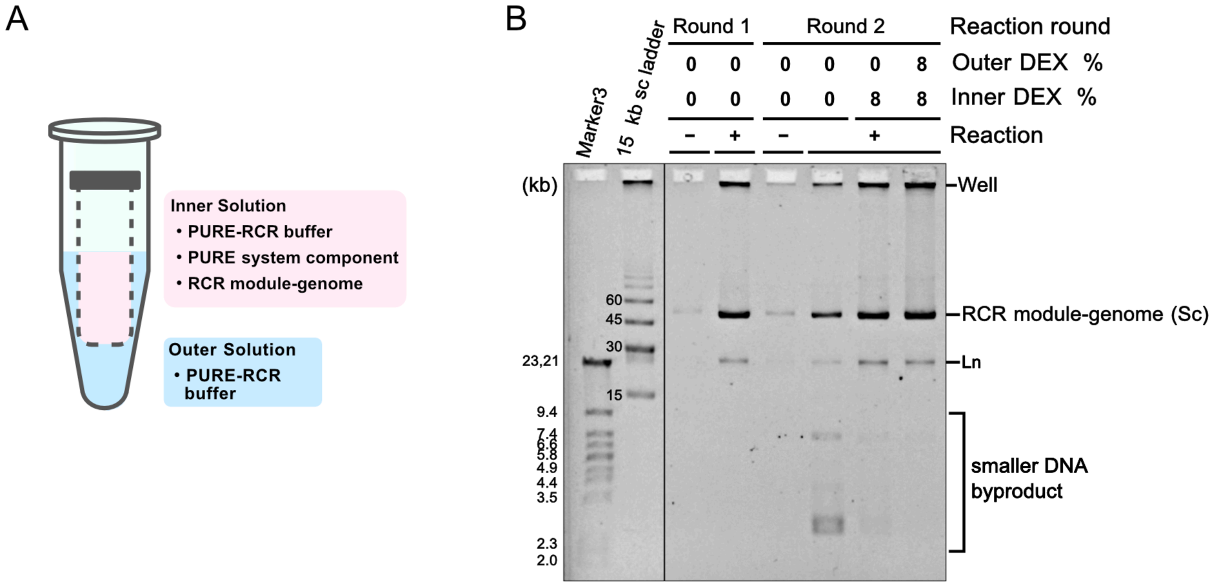

Supplementary Figure 7 Suppression of non-specific byproducts in the dialyzed PURE-RCR system.

Agarose gel electrophoresis showing the reduction of non-specific byproducts upon addition of dextran (DEX). Self-replication was performed in Round 1 using 100 pM RCR module-genome v5, and the amplified products were diluted to 100 pM for Round 2. DEX was added to the inner and outer solutions at concentrations of 0% or 8%. The addition of DEX to both inner and outer solutions suppressed the amplification of non-specific byproducts smaller than 15 kb, enabling selective self-replication of the RCR module-genome. Bands indicate supercoiled (sc), open circular (oc), linear (ln), concatemer (Cmer), and replication intermediates in the well (Well).

Alt text: Schematic of the dialyzed PURE-RCR system and an agarose gel image showing that dextran supplementation suppresses smaller DNA byproducts during serial self-replication of the RCR module-genome.

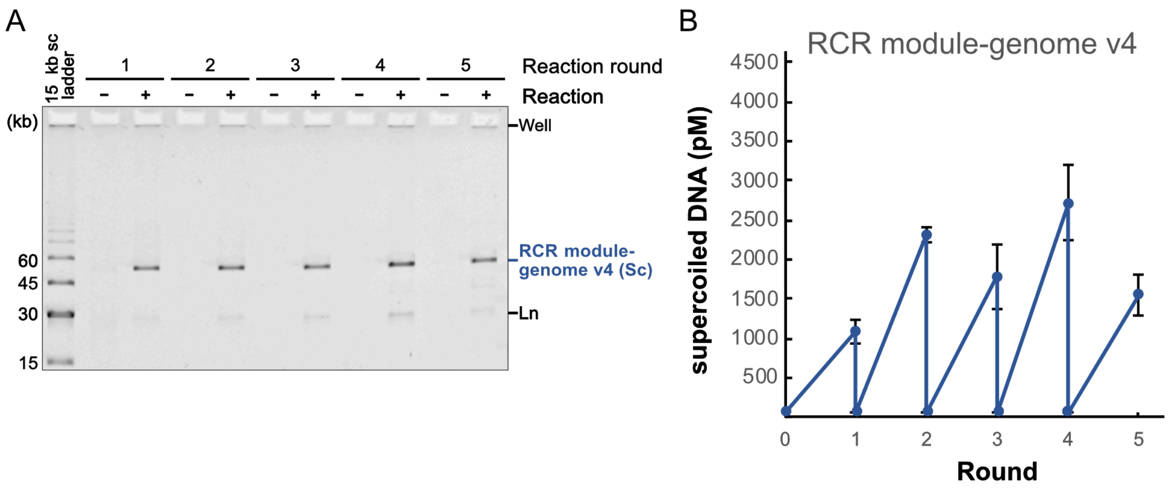

Supplementary Figure 8 Achievement of recursive self-replication using the RCR module-genome v4.
(A) Results of the serial transfer experiment. Starting from the 50 pM RCR module-genome v4, amplification was sustained up to round 5. **(B)** Quantification of RCR module-genome v4 amplification during the serial transfer experiment. The amount of amplified DNA was calculated from the band intensity of the supercoiled (sc) form. Data are presented as mean ± SD (n=3). All agarose gel images in this figure show reaction products separated on a 0.5% agarose gel (60 V, 60 min) and stained with SYBR Green I. The leftmost lane contains a DNA size marker (15 kb supercoil ladder). Labels on the right indicate supercoiled (sc) and linear (ln) forms of the RCR module-genome.

Alt text: Agarose gel image and line graph showing serial transfer self-replication of RCR module-genome v4 sustained over five rounds, achieving 26 doublings (2^{26}-fold amplification) of recursive amplification.

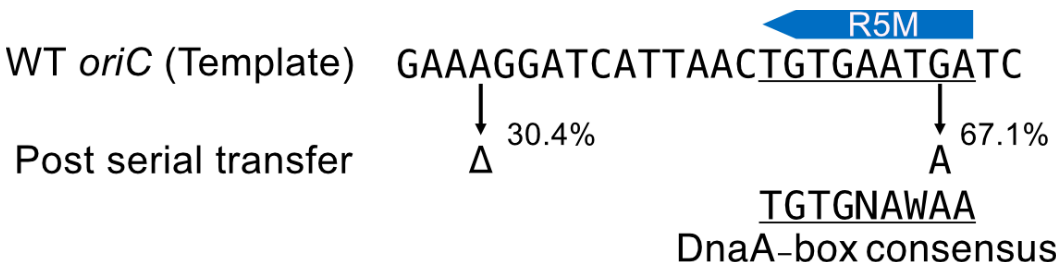

Supplementary Figure 9 Sequence changes detected in the final serial-transfer product. The sequence surrounding the detected *oriC* variant is shown together with the wild-type *oriC* sequence (the input DNA template). Two major changes detected in the final serial-transfer product are shown with their frequencies.

Alt text: Sequence comparison of the wild-type *oriC* region and the final serial-transfer product, showing two major sequence changes detected near the R5M DnaA-box motif with their observed frequencies.

POI	name	DNA concentration used in experiments	ref
DnaA	pET-dnaA	12.7 ng/µL	gbk in Zip file
IhfA, IhfB	pET-ihfB-ihfA	13.2 ng/µL	(27)
DnaB, DnaC	pET-dnaC-dnaB	11.9 ng/µL	(27)
DnaG	pET-dnaG	8.5 ng/µL	(27)
GyrA	pET-gyrA	9.4 ng/µL	(27)
GyrB	pET-his-gyrB	12.9 ng/µL	(27)
DnaN	pET-dnaN	6.8 ng/µL	(27)
Poll III star	pET-polIII*	11.0 ng/µL	(27)
Pol I	pET-polI	9.9 ng/µL	gbk in Zip file
SSB	pET-ssb	14.0 ng/µL	(27)
Ligase	pET-his-ligA	9.3 ng/µL	gbk in Zip file
Topo III	pET-his-topB	10.5 ng/µL	(27)
RecQ	pET-his-recQ	3.5 ng/µL	(27)
RNaseH	pET-rnhA	2.8 ng/µL	gbk in Zip file
ParCE	pETcoco-parC, pET-parE	3.7 ng/µL each	(27)
Tus	pET-his-tus	3.0 ng/µL	(27)
Reporter (oriC-16k)	pLter15kb_ter	0.5 ng/µL	gbk in Zip file

Supplementary Table 1 List of pET plasmid constructs and reporter plasmids for rescue assay
Detailed information on expression plasmid and reporter plasmid constructs used in Figure 1. Includes POI, plasmid name, input DNA concentration (ng/µL) and reference. Complete sequences of operon plasmids are provided as GenBank files in a supporting zip file.

No.	name	construct	purpose
1001	pT7hyb-DCDB-ihfBA*1	TT7hyb10-PAmp-AmpR-pUCori-bom-rop-LacI-Plac-PT7-dnaC-dnaB-ihfB-ihfA	For construction of RCR module-genome v1, 2, 3, 4, 5
1002	pT7hyb-gA-DG	TT7hyb10-PAmp-AmpR-pUCori-bom-rop-LacI-Plac-PT7-gyrA-dnaG	For construction of RCR module-genome v1
1003	pT7hyb-DA-gB	TT7hyb10-PAmp-AmpR-pUCori-bom-rop-LacI-Plac-PT7-dnaA-gyrB	For construction of RCR module-genome v1, 2, 3, 4, 5
1004	pT7hyb-hCDBA-DX*2	TT7hyb10-PAmp-AmpR-pUCori-bom-rop-LacI-Plac-PT7-holC-holD-holB-holA-dnaX	For construction of RCR module-genome v1, 2, 3, 4, 5
1005	pT7hyb-DQ-HE-DE*3	TT7hyb10-PAmp-AmpR-pUCori-bom-rop-LacI-Plac-PT7-dnaQ-holE-dnaE	For construction of RCR module-genome v1, 2, 3, 4, 5
1006	pT7hyb-LA-TB	TT7hyb10-PAmp-AmpR-pUCori-bom-rop-LacI-Plac-PT7-ligA-TopB	For construction of RCR module-genome v1, 2, 3, 4, 5
1007	pT7hyb-SSB-PolA-DnaN	TT7hyb10-PAmp-AmpR-pUCori-bom-rop-LacI-Plac-PT7-ssb-polA-dnaN	For construction of RCR module-genome v1, 2, 3
1008	pT7hyb-rQ-rnh-tus	TT7hyb10-PAmp-AmpR-pUCori-bom-rop-LacI-Plac-PT7-recQ-rnhA-tus	For construction of RCR module-genome v1, 2, 3, 4, 5
1009	pT7hyb-pCpE*4	TT7hyb10-PAmp-AmpR-pUCori-bom-rop-LacI-Plac-PT7-parC-parE	For construction of RCR module-genome v1, 2, 3, 4, 5
1010	pT7hyb-GyrA-SKDG	TT7hyb10-PAmp-AmpR-pUCori-bom-rop-LacI-Plac-PT7-gyrA-skik_dnaG	For construction of RCR module-genome v2, 4
1011	pT7hyb-SKDG-GA	TT7hyb10-PAmp-AmpR-pUCori-bom-rop-LacI-Plac-PT7-skik_dnaG-gyrA	For construction of RCR module-genome v3, 5
1012	pT7hyb-SKSSB-PolA-DnaN	TT7hyb10-PAmp-AmpR-pUCori-bom-rop-LacI-Plac-PT7-SKIK_ssb-polA-dnaN	For construction of RCR module-genome v4, 5
1013	oriC-4k	*oriC*	For Reporter Assay

Supplementary Table 2 List of operon plasmid constructs and reporter plasmids
Detailed information on all operon plasmid and reporter plasmid constructs used in this study. Includes plasmid No., name, encoded genes and purpose. Complete sequences of operon plasmids are provided as GenBank files in a supporting zip file. *1–*4 indicate constructs encoding proteins that form functional complexes. *1, DnaB–DnaC and IhfA–IhfB. *2, HolA–HolB–HolC–HolD–DnaX. *3, DnaE–DnaQ–HolE. *4, ParC–ParE.

No	name	Fwd Primer	Rev Primer	Template No.	Method
Fr001	oriC2.61vec_for_iE(001-004)				
Fr002	oriC2.61vec_for_eE(005-009)				
Fr003	oriC2.61vec_for_sE(010-012)				
Fr004	101-Spacer1-103				
Fr005	15-Spacer2-14				
Fr006	004-DCDB-ihfBA-101	SUE12934	SUE13106	1001	PCR 2step
Fr007	103-gA-DG-002	SUE12931	SUE13107	1002	PCR 2step
Fr008	002-DA-gB-001	SUE12929	SUE12930	1003	PCR 2step
Fr009	005-hCDBA-DX-006	SUE12937	SUE12938	1004	PCR 2step
Fr010	006-DQ-HE-DE-015	SUE12939	SUE13105	1005	PCR 2step
Fr011	014-LA-TB-008	SUE13104	SUE12942	1006	PCR 2step
Fr012	008-ssb-polA-dnaN-009	SUE12944	SUE12943	1007	PCR 2step
Fr013	010-RQ-RN-tus-011	SUE12955	SUE12956	1008	PCR 2step
Fr014	011-PC-PE-012	SUE12957	SUE12958	1009	PCR 2step
Fr015	103-gA-skik_DG-002	SUE12931	SUE13107	1010	PCR 2step
Fr016	103-skik_DG-gA-002	SUE12931	SUE13107	1011	PCR 2step
Fr017	008-skik_ssb-polA-dnaN-009	SUE12944	SUE12943	1012	PCR 2step
Fr018	iEv2.1-AvrII			1101	AvrII cut
Fr019	eEv2.1-AvrII			1102	AvrII cut
Fr020	sEv1.1-AvrII			1103	AvrII cut
Fr021	iEv3.1-AvrII			1104	AvrII cut
Fr022	iEv4.1-AvrII			1105	AvrII cut
Fr023	eEv3.1-AvrII			1106	AvrII cut

Supplementary Table 3 List of PCR fragments
Information on PCR fragments used for construction of Sub-module-DNAs and RCR module-genome. Includes name, forward primer (Fwd Primer), reverse primer (Rev Primer), template number (Template No.), and fragmentation method (Method) for each fragment. Complete sequences of Fr001, 002, 003, 004, 005 are provided as GenBank files in a supporting zip file.

No.	name	DNA fragment (FrXXX)	Cloning Method
1101	iE sub-module-DNA v2.1	001, 006, 004, 007, 008	Assembly-RCR
1102	eE sub-module-DNA v2.1	002, 009, 010, 005, 011, 012	Assembly-RCR
1103	sE sub-module-DNA v1.1	003, 013, 014	Assembly-RCR
1104	iE sub-module-DNA v3.1	001, 006, 004, 015, 008	Assembly-RCR
1105	iE sub-module-DNA v4.1	001, 006, 004, 016, 008	Assembly-RCR
1106	eE sub-module-DNA v3.1	002, 009, 010, 005, 011, 017	Assembly-RCR
1201	RCR module-genome v1	018, 019, 020	Assembly-RCR
1202	RCR module-genome v2	021, 019, 020	Assembly-RCR
1203	RCR module-genome v3	022, 019, 020	Assembly-RCR
1204	RCR module-genome v4	021, 023, 020	Assembly-RCR
1205	RCR module-genome v5	022, 023, 020	Assembly-RCR

Supplementary Table 4 Detailed information on Sub-module-DNAs and RCR module-genome

Detailed information on the constructed Sub-module-DNAs (iE, eE, sE) and RCR module-genome. Includes name, DNA fragments used (DNA fragment, FrXXX format), and cloning method (Cloning Method) for each DNA construct.

Primer No	Sequence
SUE12929	ACTGACCCCACAAGGCCCTAGCGTTTGCAATGCACCAGGTGATCTTCCCCATCG GTGATGTCG
SUE12930	AGCCTAAGTTTACACAAGTTATACGGTCACACGGGCAGCGCCCATTCGCCAATCC GGATATAGTTCC
SUE12931	CGCTGCCCGTGTGACCGTATAACTTGTGTAAACTTAGGCTGATCTTCCCCATCGG TGATGTCG
SUE12934	tactcattatagtgtgaaaatgaccctctgtcaacagctcGATCTTCCCCATCGGTGATGTCG
SUE12937	accatcatcagtgaaccggaccgtaatgtccgctatgcccGATCTTCCCCATCGGTGATGTCG
SUE12938	GACTGTTACCTACGACCTGCACGAACACGCCAGGCACACGCCCATTCGCCAATC CGGATATAGTTCC
SUE12939	CGTGTGCCTGGCGTGTTCGTGCAGGTCGTAGGTAACAGTCGATCTTCCCCATCG GTGATGTCG
SUE12942	CAGTCGCAGTCCTTGGGTTGATGTTAACTCTCGCATTTGGGATCTTCCCCATCGG TGATGTCG
SUE12943	CCAAATGCGAGAGTTAACATCAACCCAAGGACTGCGACTGCCCATTCGCCAATCC GGATATAGTTCC
SUE12944	cgttctccagcttcagtgccgtgaacggtgaatcgctcacGATCTTCCCCATCGGTGATGTCG
SUE12955	cgttcctttctgtacggcattgttaatggcatccgtccagGATCTTCCCCATCGGTGATGTCG
SUE12956	AATGGTCCGTATTCAACTAATGATCGCGTCTGTTTTCCCGCCCATTCGCCAATCCG GATATAGTTCC
SUE12957	CGGGAAAACAGACGCGATCATTAGTTGAATACGGACCATTGATCTTCCCCATCGG TGATGTCG
SUE12958	ctgggagacacatcgggacgcacttgtcctggtggataatCCCATTCGCCAATCCGGATATAGTTCC
SUE13104	agtagtggttcacgggccagccatgatgctcgtcatgataCCCATTCGCCAATCCGGATATAGTTCC
SUE13105	cataaaggcggcaatggcgttcaggttattccgcaaccacCCCATTCGCCAATCCGGATATAGTTCC
SUE13106	cagatgaaattgtccgtaaaatggcagagaacaaacctgaCCCATTCGCCAATCCGGATATAGTTC C
SUE13107	cccttctcacggtcgagggggataagctgctcacggctgaCCCATTCGCCAATCCGGATATAGTTCC

Supplementary Table 5 List of primer sequences
Sequence information for all primers used in the construction of Sub-module-DNAs. Includes primer name and sequence (5'→3').

T5 buffer component	concentration
Tween20	0.05% (w/v)
Hepes-KOH (pH 7.6)	50 mM
DTT	2 mM
KGlu	75 mM
spermidine	0.2 mM
Mg(oAc)2	15 mM
Tiron	0.1 mM
Creatine Phosphate	20 mM
ATP	2 mM
GTP	2 mM
CTP	1 mM
UTP	1 mM
tRNA (*E. coli*)	1 mg/mL
Folinic Acid Calcium Salt	0.01 mg/mL
NAD+	50 µM
dNTPs	0.1 mM each

Supplementary Table 6 T5 buffer components

Triplicate		Round 1	2	3	4	5
#1	SC product (pM)	2013.0	2287.2	2245.7	3266.9	3167.4
	Fold dilution for subsequent reaction	40.3	45.7	44.9	65.3	63.3
#2	SC product (pM)	2029.5	2155.0	2691.1	4319.6	2606.1
	Fold dilution for subsequent reaction	40.6	43.1	53.8	86.4	52.1
#3	SC product (pM)	1406.3	2839.7	1469.8	3085.2	3195.0
	Fold dilution for subsequent reaction	28.1	56.8	29.4	61.7	63.9

Supplementary Table 7 Quantification of supercoiled product and fold amplification/dilution in serial transfer experiments of the RCR module-genome v5. Three independent serial transfer experiments of RCR module-genome v5 (#1, #2, and #3) were performed, each consisting of five consecutive reaction rounds. After each reaction round, the concentration of the supercoiled product (pM) was quantified, and an aliquot of the unpurified reaction mixture was directly diluted into fresh PURE reaction solution to initiate the next round. The "Fold Amplification, Fold dilution" value represents the ratio between the supercoiled product concentration at the end of a round and the input DNA concentration of that round; because the diluted aliquot was used as the input for the subsequent round, this value is equivalent to the dilution fold applied at each transfer. The data shown here are the source values used to generate Figure 4g. SC indicates the supercoiled DNA replication product.

Triplicate		Round 1	2	3	4	5
#1	SC product (pM)	1248	2203	1911	3218	1676
	Fold dilution for subsequent reaction	25.0	44.1	38.2	64.4	33.5
#2	SC product (pM)	974	2336	1336	2598	1239
	Fold dilution for subsequent reaction	19.5	46.7	26.7	51.9	24.8
#3	SC product (pM)	1034	2382	2126	2281	1705
	Fold dilution for subsequent reaction	20.7	47.6	42.5	45.6	34.1

Supplementary Table 8 Quantification of supercoiled product and fold amplification/dilution in serial transfer experiments of the RCR module-genome v4. Three independent serial transfer experiments of RCR module-genome v4 (#1, #2, and #3) were performed, each consisting of five consecutive reaction rounds. After each reaction round, the concentration of the supercoiled product (pM) was quantified, and an aliquot of the unpurified reaction mixture was directly diluted into fresh PURE reaction solution to initiate the next round. The "Fold Amplification, Fold dilution" value represents the ratio between the supercoiled product concentration at the end of a round and the input DNA concentration of that round; because the diluted aliquot was used as the input for the subsequent round, this value is equivalent to the dilution fold applied at each transfer. The data shown here are the source values used to generate Supplementary Figure 8. SC indicates the supercoiled DNA replication product.

RCR module-genome version	gene	RBS sequence	Native or pET
v1	DnaA	AAGAAGGAGA	pET
v1	GyrA	AAGAAGGAGA	pET
v1	GyrB	AAGAAGGAGA	pET
v1	DnaG	AAGAAGGAGA	pET
v1	DnaC	AAGAAGGAGA	pET
v1	DnaB	AAGGAGG	Optimized
v1	IhfB	AAGAAGGAGA	pET
v1	IhfA	GAGGGA	Native MG1655
v1	HolC	AAGAAGGAGA	pET
v1	HolD	AGGAGG	Optimized
v1	HolB	AGGAGG	Optimized
v1	HolA	AGGAGG	Optimized
v1	DnaX	AGGAGG	Optimized
v1	DnaQ	AAGAAGGAGA	pET
v1	HolE	AGGAGA	Native MG1655
v1	DnaE	AGGAGAA	Optimized
v1	SSB	AAGAAGGAGA	pET
v1	PolA	GAAGGAGA	Optimized
v1	DnaN	AAGAAGGAGA	pET
v1	LigA	AAGAAGGAGA	pET
v1	TopB	AAGAAGGAGA	pET
v1	RecQ	AAGAAGGAGA	pET
v1	RnhA	AAGAAGGAGA	pET
v1	Tus	AAGAAGGAGA	pET
v1	ParC	AAGAAGGAGA	pET

v1	ParE	AAGAAGGAGA	pET
v2, v4	GyrA	AAGAAGGAGA	pET
v2, v4	SKIK_DnaG(CO)	AAGAAGGAGA	pET
v3, v5	SKIK_DnaG(CO)	AAGAAGGAGA	pET
v3, v5	GyrA	AAGAAGGAGA	pET

Supplementary Table 9. RBS sequences used in RCR module-genomes.
Ribosome binding site (RBS) sequences used for each gene in the RCR module-genomes are listed. The table indicates the corresponding RCR module-genome version, gene name, RBS sequence, and whether the RBS was derived from the pET vector, the native *E. coli* MG1655 sequence, or an optimized sequence.

Uncropped gel image

Fig1. C

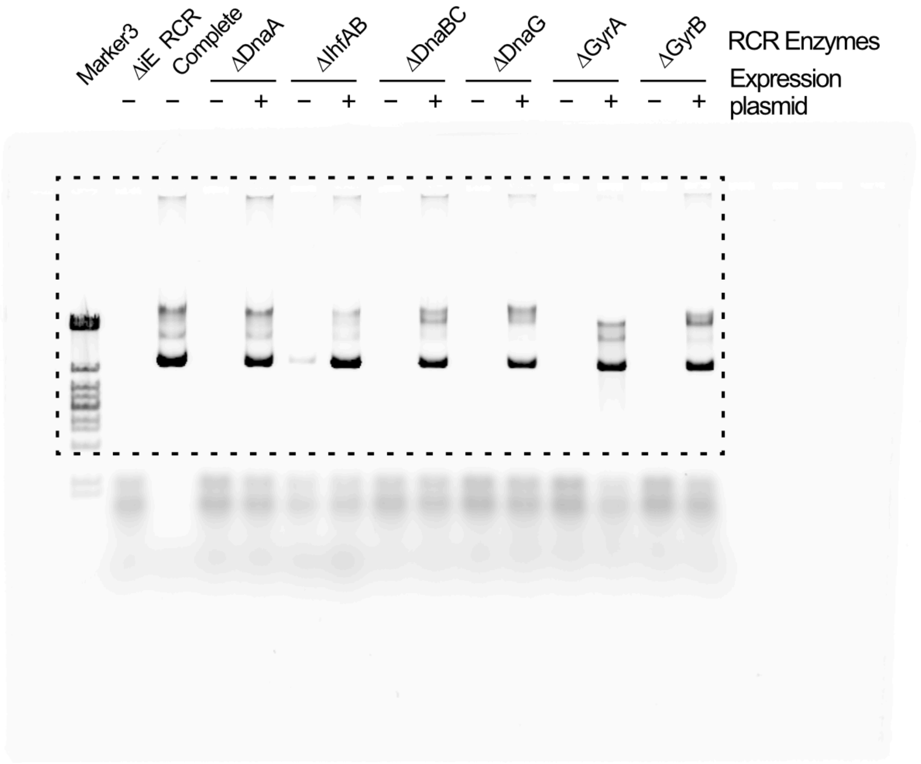

Fig1. D

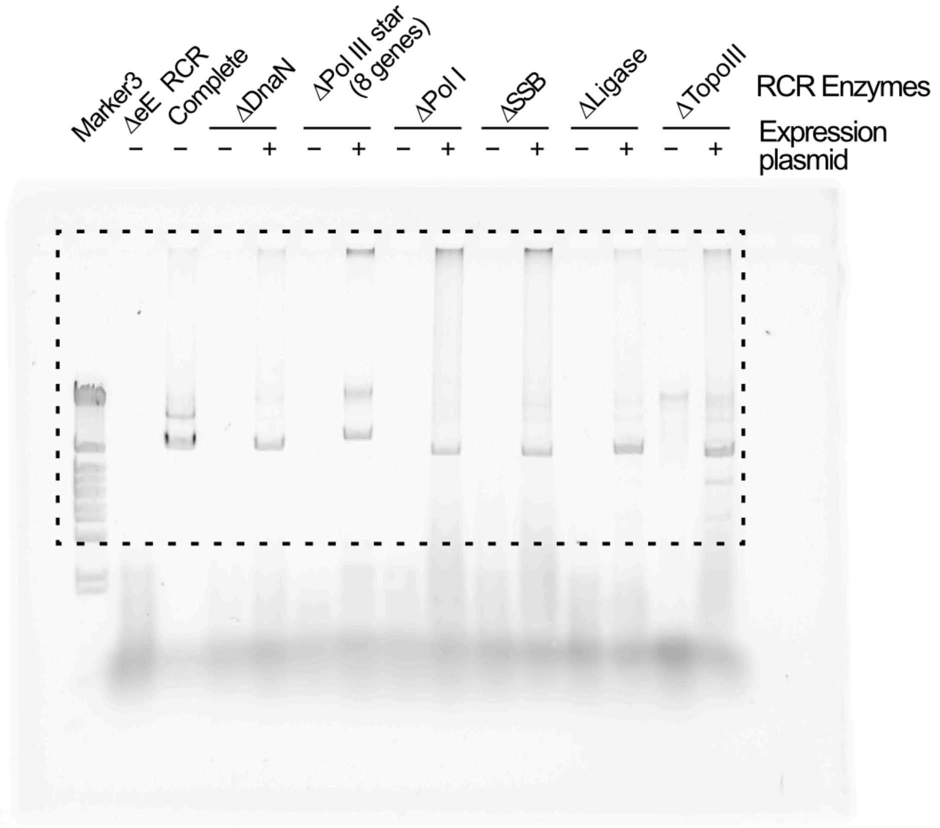
Marker3
ΔeE RCR
Complete
ΔDnaN
ΔPol III star (8 genes)
ΔPol I
ΔSSB
ΔLigase
ΔTopoIII
RCR Enzymes
Expression plasmid
− − − + − + − + − + − + − +

Fig1. E

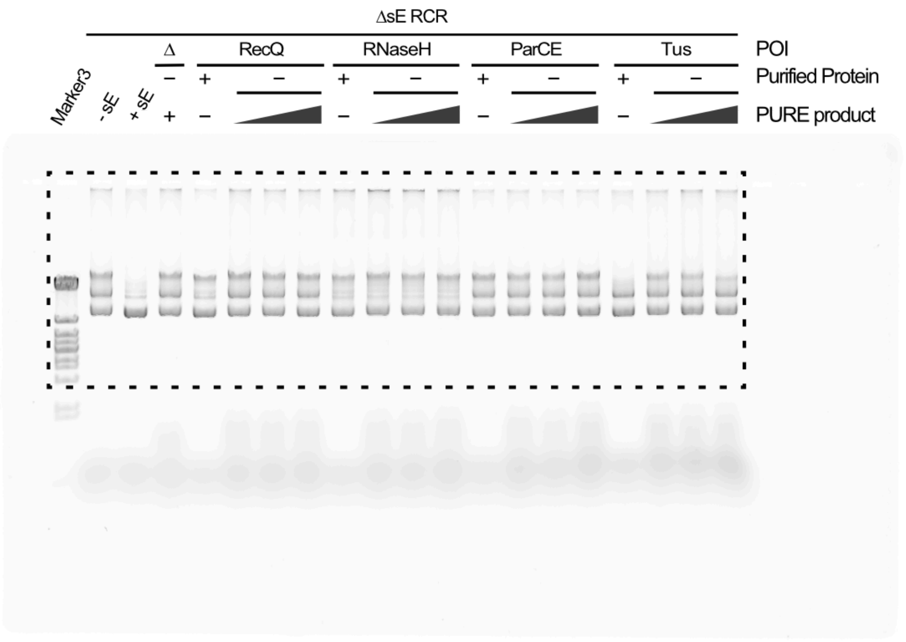
ΔsE RCR
Marker3
- sE
+ sE
Δ
RecQ
RNaseH
ParCE
Tus
POI
Purified Protein
PURE product

Fig2. C

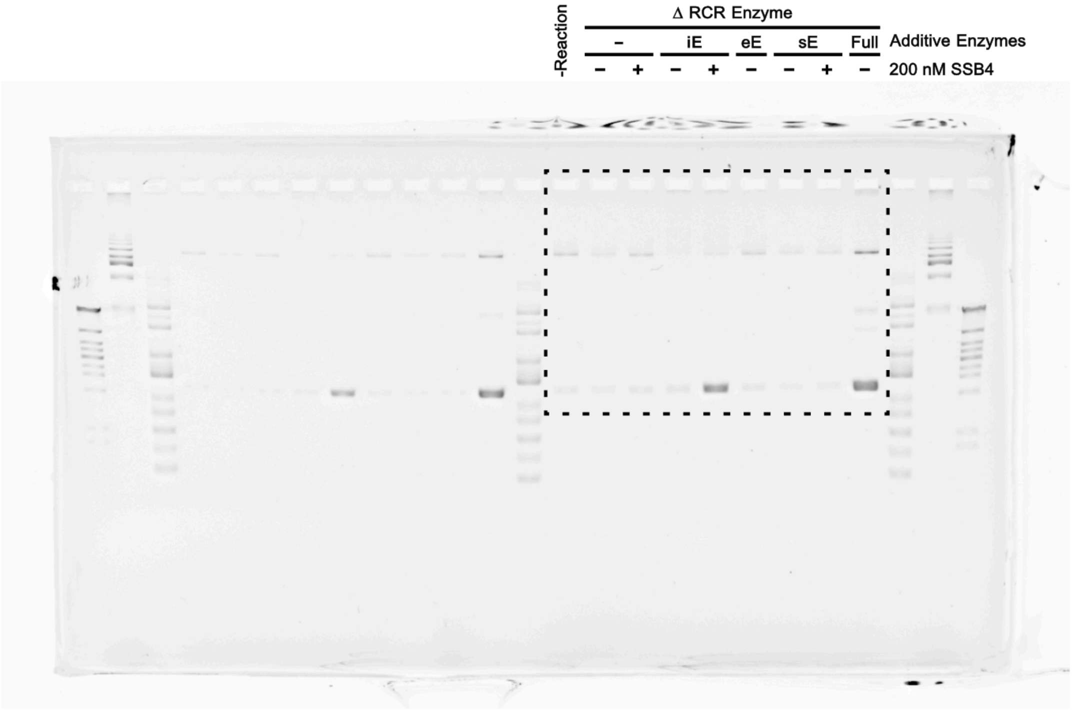

-Reaction
Δ RCR Enzyme
– iE eE sE Full Additive Enzymes
– + – + – – + – 200 nM SSB4

Fig3. B

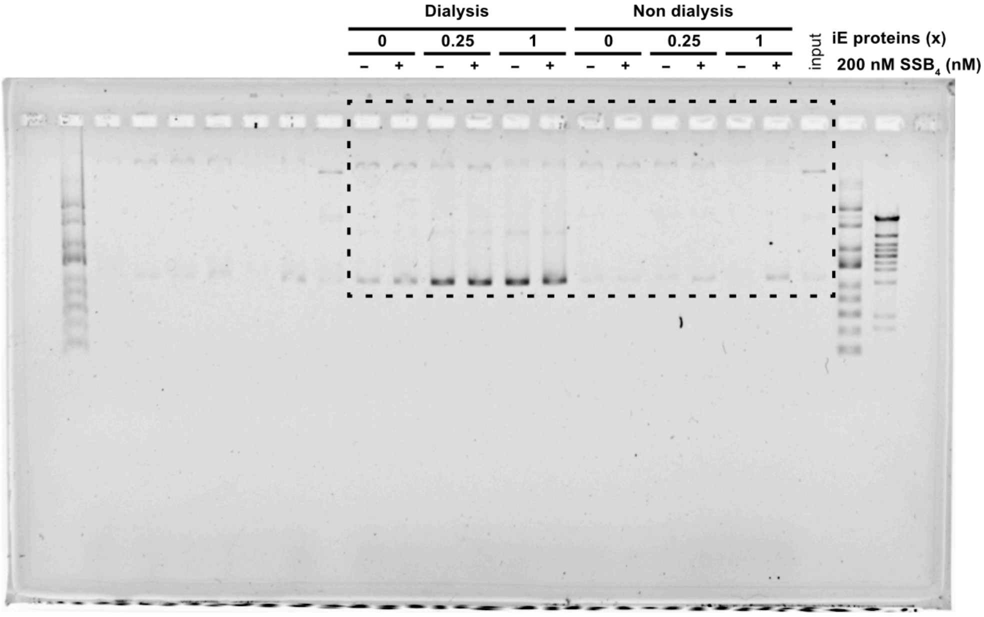

Dialysis
Non dialysis
0 0.25 1 0 0.25 1 iE proteins (x)
– + – + – + – + – + – + 200 nM SSB_4 (nM)
input

Fig3. C

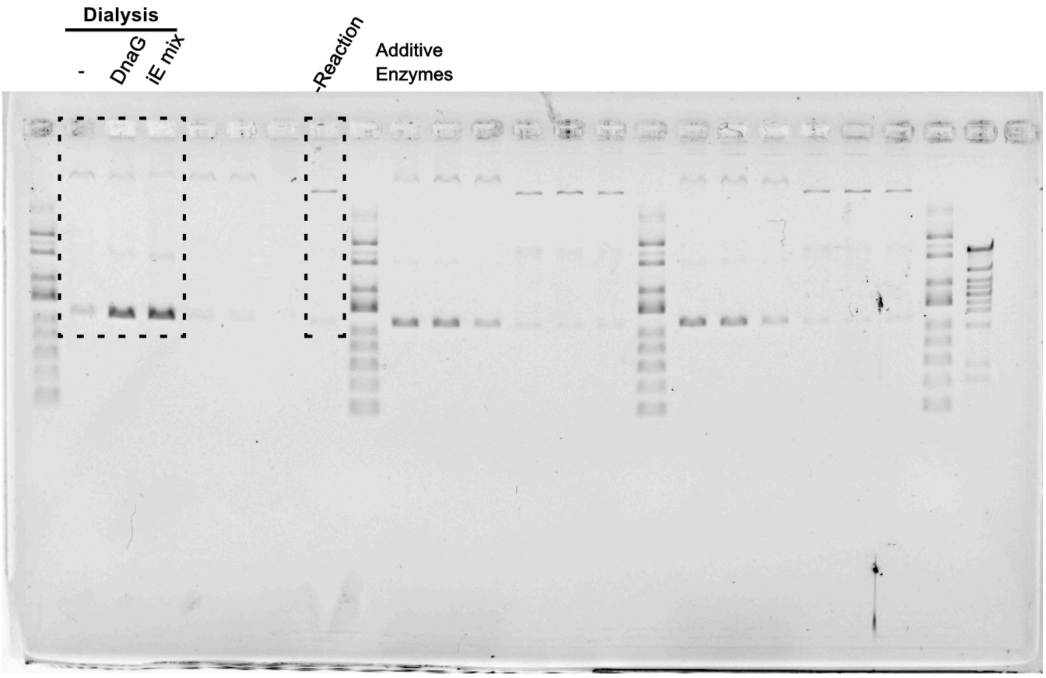
Dialysis
-
DnaG
iE mix
-Reaction
Additive
Enzymes

Fig3. E

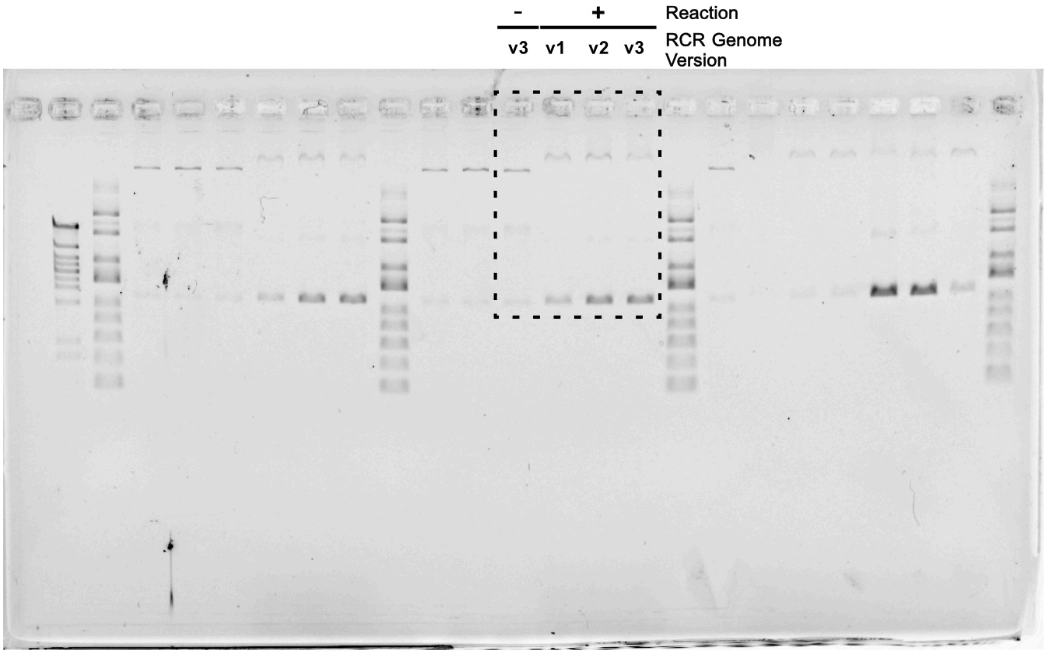
-
+
Reaction
v3
v1
v2
v3
RCR Genome
Version

Fig4. B

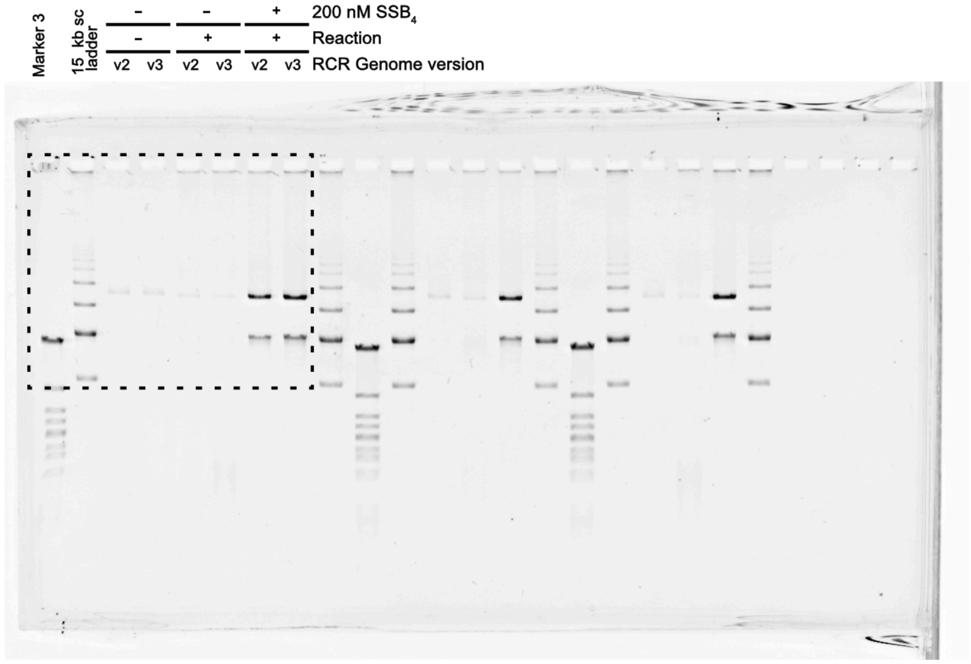
Marker 3
15 kb sc ladder
– – + 200 nM SSB_4
– + + Reaction
v2 v3 v2 v3 v2 v3 RCR Genome version

Fig4. D

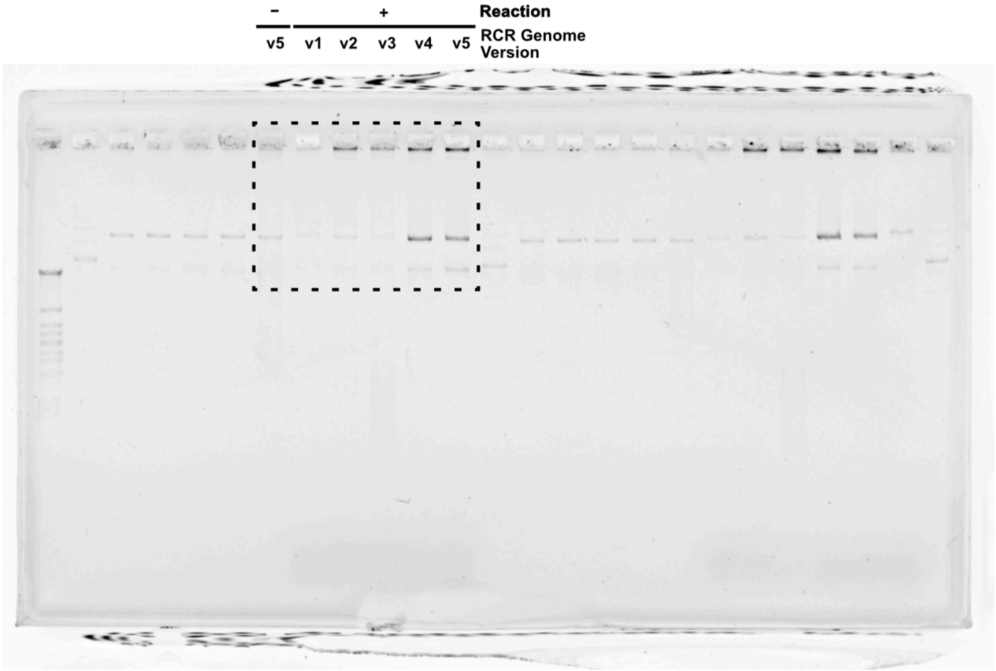
– + Reaction
v5 v1 v2 v3 v4 v5 RCR Genome Version

Fig4. F

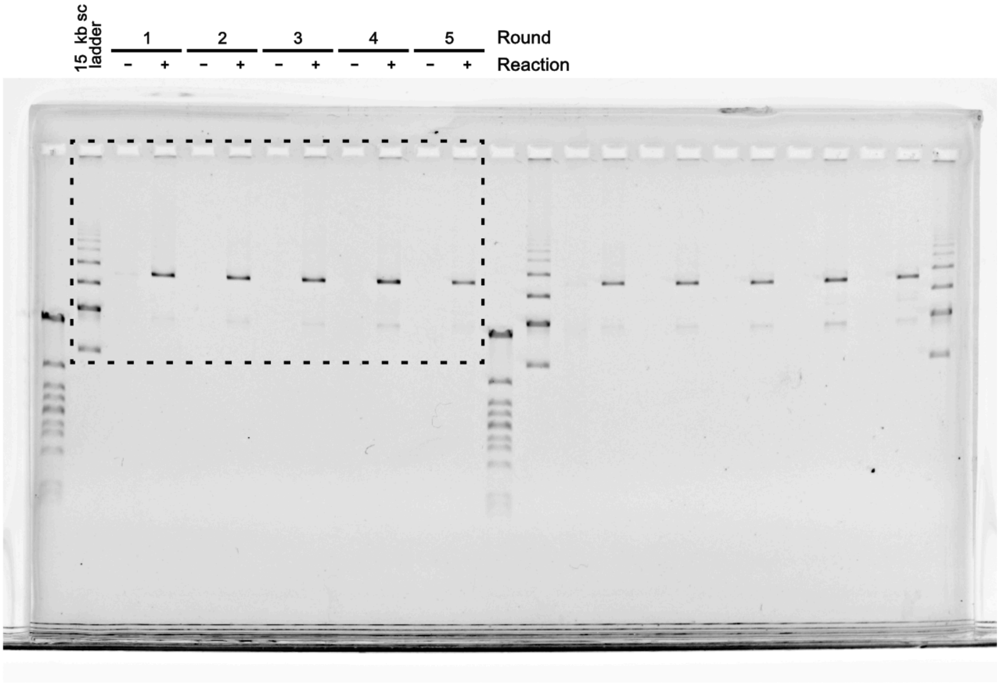

FigS8. A

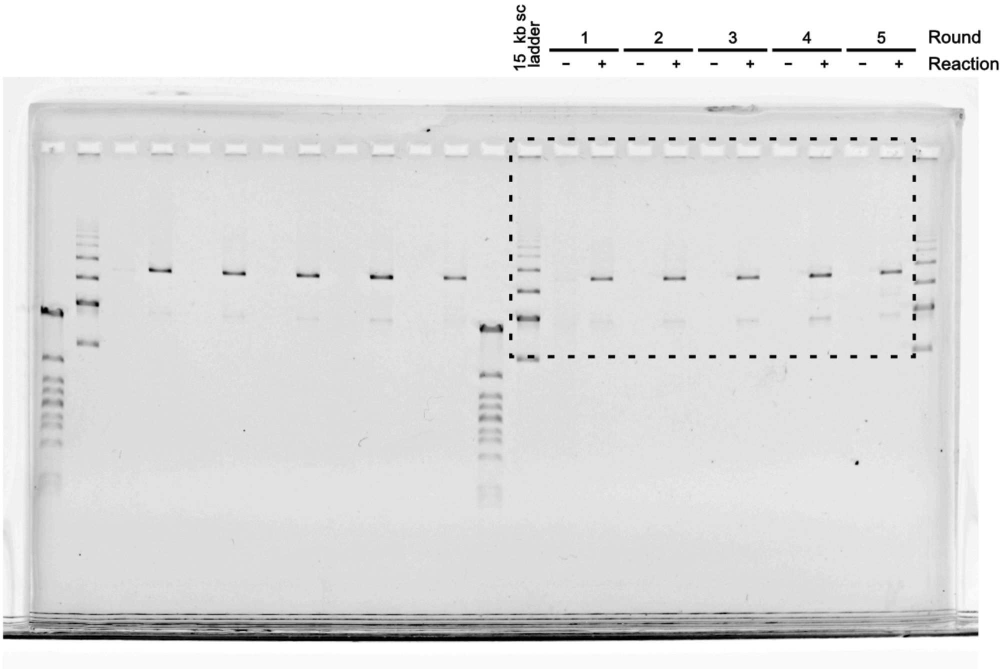

Fig4. G
FigS8. B
Round 1

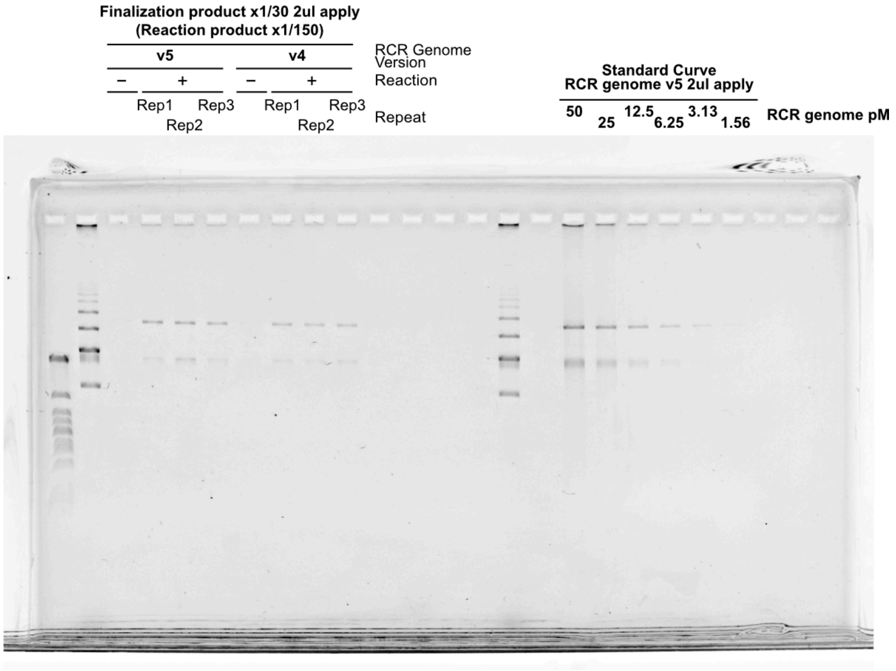

Fig4. G
FigS8. B
Round 2

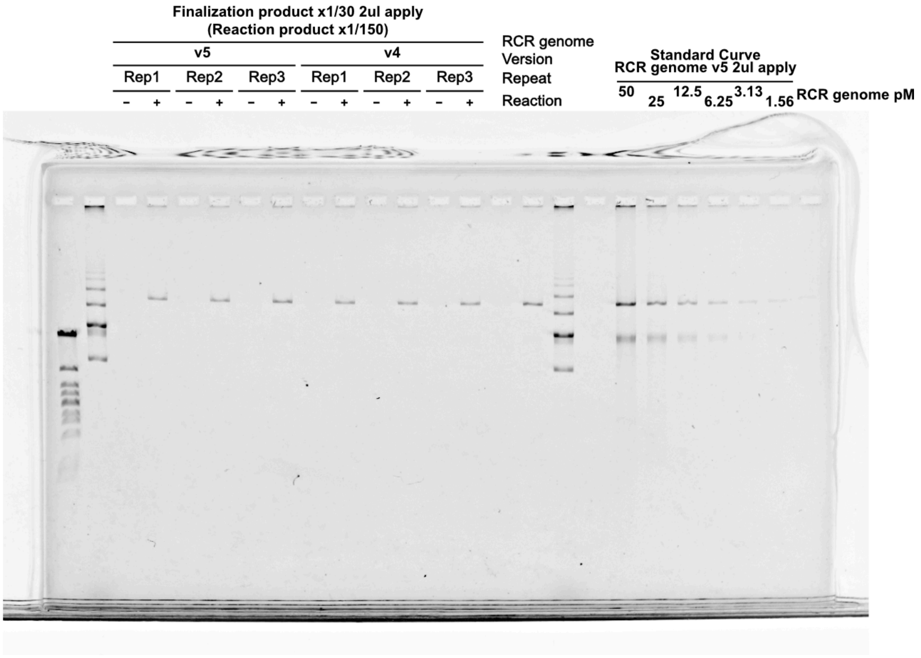

Fig4. G
FigS8. B
Round 3

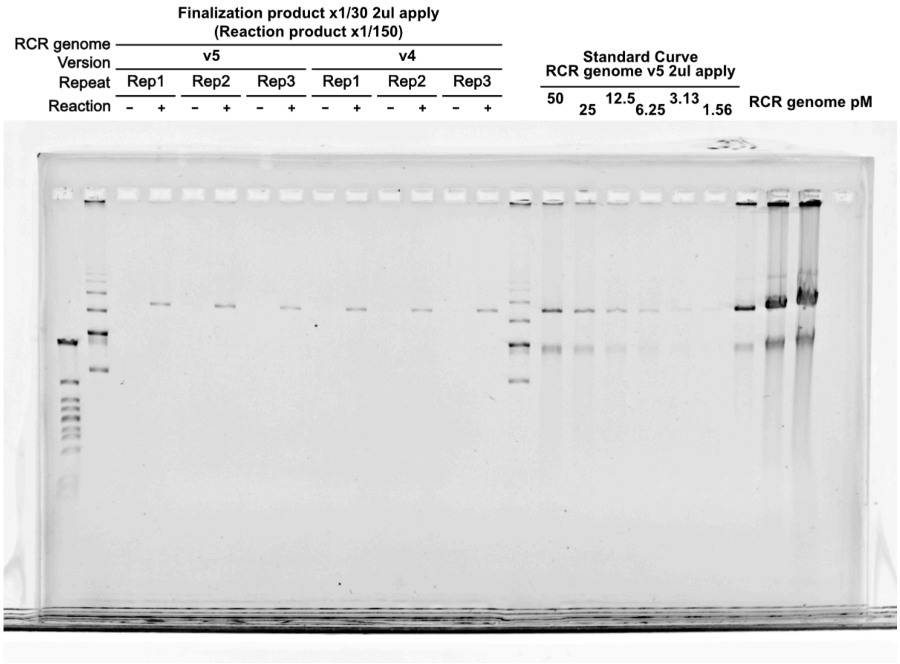

Fig4. G
FigS8. B
Round 4

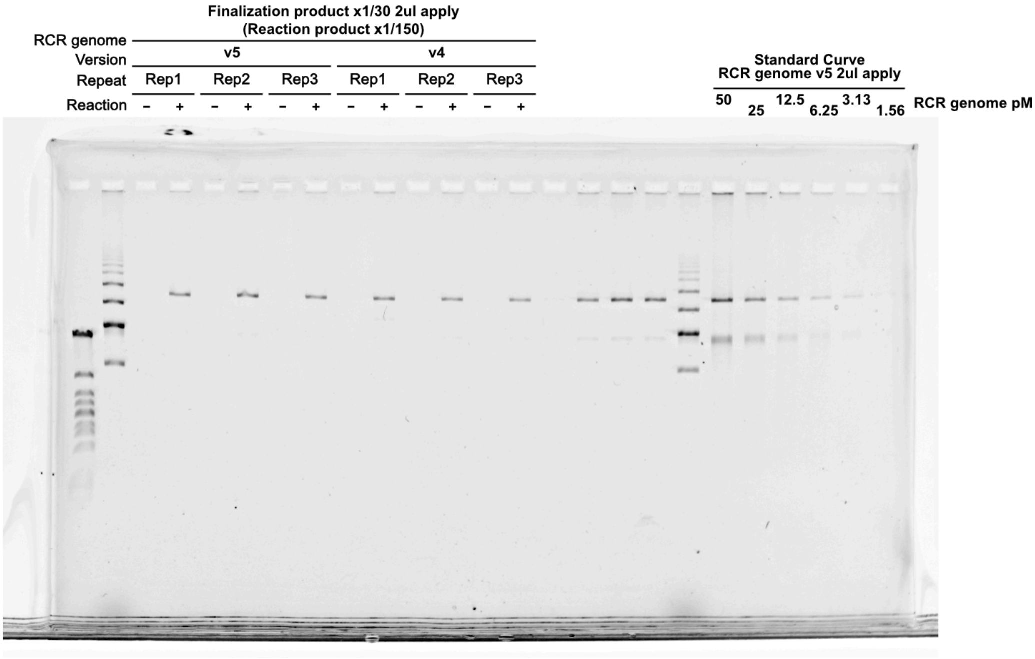

Fig4. G
FigS8. B
Round 5

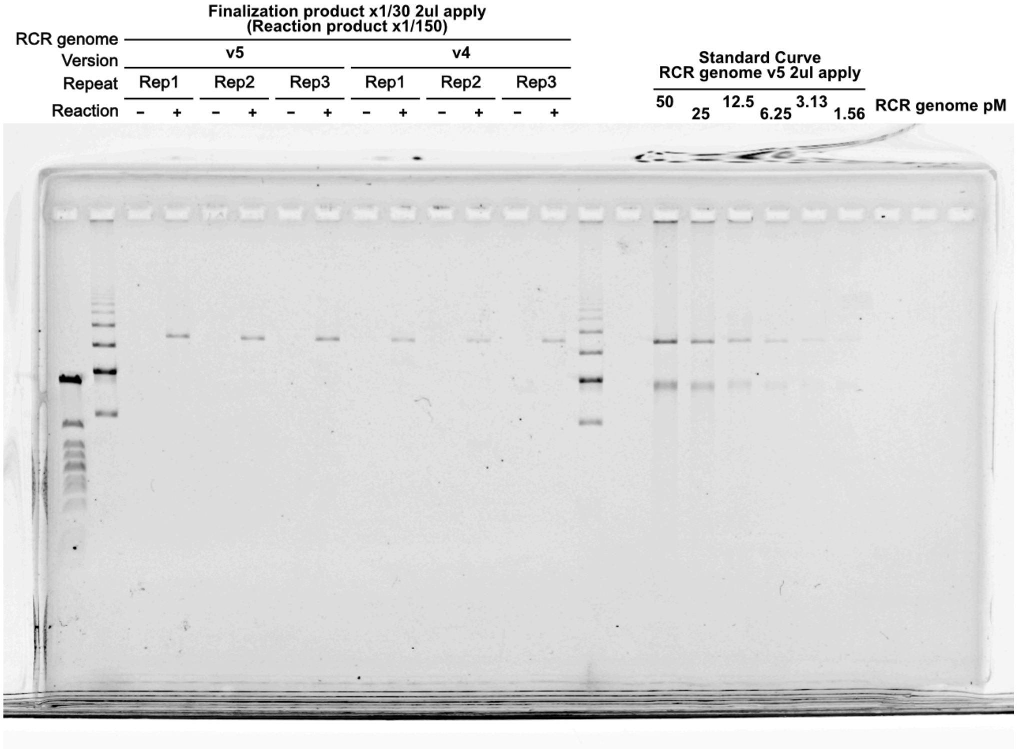

FigS7. B

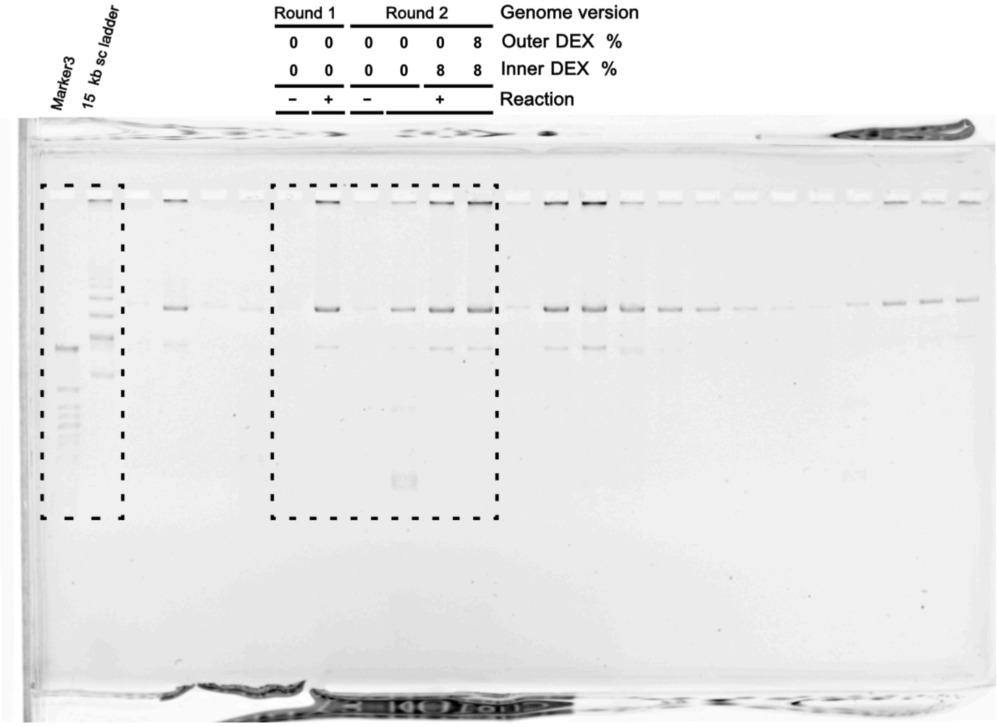

Supplement: gkag663_Supplemental_Files [file gkag663_supplemental_files.zip › Supplementalt Material_NAR_revise.pdf]
